# Multiscale cross modal fusion and cascaded signal propagation for drug target interaction prediction

**DOI:** 10.1016/j.isci.2026.117576

**Published:** 2026-09-22

**Authors:** Bowen Wang, Xiaolan Xie, Haitao Zou, Shaoliang Peng

**Affiliations:** 1 College of Computer Science and Engineering, Guilin University of Technology, Guilin 541006, China; 2 College of Computer Science and Electronic Engineering, Hunan University, Changsha 410082, China

**Keywords:** drug-target interaction prediction, multiscale fusion, subspace alignment, chemical language model, protein language model

## Abstract

Accurate prediction of drug-target interaction (DTI) facilitates the translation of lead compounds into clinical therapeutics. Despite progress in deep learning, two challenges remain: the representation gap between heterogeneous drug and protein modalities and insufficient use of complementary knowledge from pretrained encoders. To address these issues, we propose SF-DTI, a unified framework that integrates multi-encoder protein fusion, structure-semantic drug alignment, and progressive cross-modal refinement for DTI prediction. Specifically, SF-DTI captures global protein semantics from multiple pretrained protein language models, aligns graph-based structural features with pretrained semantic embeddings for drugs under subspace constraints, and progressively refines drug-protein interaction representations through cascaded cross-modal signal propagation. Experiments on six benchmark datasets under standard, cold-start, and cross-domain settings demonstrate that SF-DTI achieves competitive performance relative to seven state-of-the-art baselines across evaluation scenarios. These results suggest that SF-DTI is a promising framework for DTI prediction and may support virtual screening, drug repositioning, and drug discovery.

## Highlights

• SF-DTI integrates molecular graphs with pretrained drug and protein semantics
• Cascaded cross-modal propagation refines shared drug-protein interaction signals
• SF-DTI performs strongly across standard, cold-start, and cross-domain settings

## Graphical abstract

**Figure:**
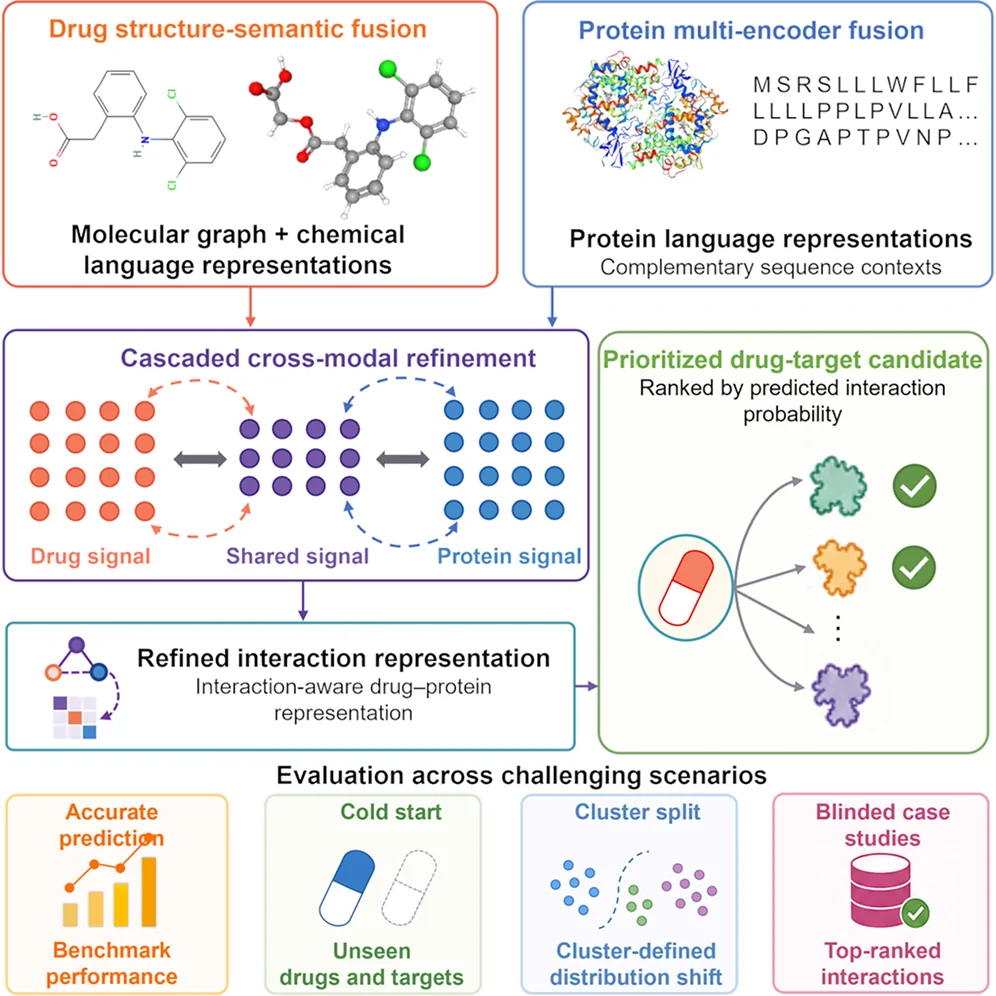

## Teaser

Protein; Bioinformatics; Computational bioinformatics; Neural networks; Artificial intelligence; Machine learning

## Introduction

Drug-target interaction (DTI) prediction is critical to drug discovery, virtual screening, and drug repurposing.^1^^,^^2^^,^^3^ Compared with costly and time-consuming experimental assays, computational DTI prediction provides an efficient strategy for prioritizing candidate compounds and reducing early-stage development costs.^4^^,^^5^ In recent years, deep learning has substantially advanced this field by enabling automatic representation learning from molecular structures and protein sequences.^6^^,^^7^ In particular, graph neural networks and multimodal learning frameworks have shown promising capabilities in modeling drug topology and integrating heterogeneous biochemical information,^8^^,^^9^^,^^10^ while pretrained protein language models (PLMs) have emerged as effective tools for capturing rich biochemical semantics from amino acid sequences.^11^^,^^12^^,^^13^^,^^14^ Despite these advances, robust DTI prediction remains challenging, especially for unseen drugs or targets and under cross-domain distribution shifts.

The effective fusion of heterogeneous drug and protein representations remains a major challenge in DTI prediction.^15^ Molecular graphs preserve atom-level topology and local structural dependencies, whereas learned semantic embeddings provide complementary global characteristics of drugs.^16^ Recent molecular prediction models have further shown that sequence-graph fusion, multimodal multi-granularity modeling, and explainable graph-based feature fusion can improve drug-related property and toxicity prediction by integrating complementary molecular views.^17^^,^^18^^,^^19^^,^^20^ However, many existing methods still combine these heterogeneous drug features through relatively generic operations, such as concatenation or shallow projection, which are often insufficient to fully exploit the complementarity between structural and semantic information.^21^^,^^22^ Protein representation learning faces a related challenge. Pretrained PLMs have become powerful tools for extracting biochemical semantics from amino acid sequences,^23^^,^^24^ and different PLMs may provide complementary biochemical cues because of their distinct architectures and pretraining schemes.^23^^,^^24^ Nevertheless, many recent DTI frameworks still rely on a single protein encoder,^25^ and even when multiple representations are available, they are typically integrated through shallow fusion rather than deep hierarchical modeling.^21^^,^^22^ As a result, existing methods remain limited in their ability to effectively integrate diverse and multiscale information across drug and protein modalities.

Beyond representation fusion, another fundamental limitation lies in interaction modeling after feature extraction. Many existing DTI methods follow a static one-pass encoding paradigm, in which drug and target features are extracted once and then directly fed into a predictor.^26^ While computationally efficient, this pipeline cannot progressively exchange and refine cross-modal signals, making it difficult to capture the subtle and dynamic compatibility patterns between molecular topology and protein context. This limitation becomes particularly critical in practical cold-start and cross-domain scenarios, where the model must generalize to previously unseen drugs or poorly characterized targets.^27^^,^^28^ Although structure-aware methods have improved interaction learning by explicitly incorporating binding-site or sub-pocket information,^29^^,^^30^ their applicability is often constrained by the availability of high-quality structural annotations or 3D data. Therefore, a more effective DTI framework should not only align heterogeneous representations more faithfully but also support iterative and interaction-aware refinement across modalities.

To address these challenges, we propose SF-DTI, a unified framework that couples heterogeneous representation alignment with cascaded cross-modal interaction refinement. Specifically, SF-DTI incorporates a multi-encoder protein mutual interaction (MPMI) module to integrate complementary semantics from multiple pretrained PLMs, a subspace-constrained mutual alignment fusion (SCMAF) module to align drug structural graphs with semantic embeddings, thereby reducing the discrepancy between heterogeneous drug representations, and a cascaded cross-modal signal propagation fusion (CMSPF) mechanism to iteratively refine interaction-aware features beyond the conventional static single-pass pipeline. Extensive experiments on six benchmark datasets demonstrate that SF-DTI achieves effective and robust performance in standard, cold-start, and cross-domain settings.

## Results

### SF-DTI improves DTI prediction across benchmark datasets

We compare SF-DTI with several representative DTI prediction models. TransformerCPI^31^ models drugs and proteins as two sequences and captures their interactions with a Transformer-style framework built on convolational neural network (CNN) and graph convolutional network (GCN) encoders but does not exploit large pretrained encoders. MolTrans^32^ decomposes drugs and proteins into substructure sequences and applies Transformer blocks for contextual representation learning, with dot-product interactions between learned substructure embeddings. More recent approaches employ richer fusion and adaptation mechanisms. DrugBAN^28^ introduces a bilinear attention network for drug-protein fusion and incorporates domain adaptation to reduce dataset shift. BINDTI^33^ proposes a bidirectional intention network to model mutual influences between drugs and proteins through attention. MADTI^34^ emphasizes multi-domain awareness and interpretability during fusion and introduces category-aware domain adaptation (CADA), which uses gradient reversal and category-specific domain discriminators to align interaction representations from labeled source-domain and unlabeled target-domain samples. By incorporating predicted interaction categories into domain alignment, CADA preserves the distinction between positive and negative DTI samples during cross-domain transfer. WDGBAN-DTI^35^ combines deep graph convolutional networks, bilinear attention, and domain-adaptation techniques to model drug-target interactions at the atomic level. MMDG-DTI^27^ further integrates multimodal feature fusion with domain generalization and is included as a recent multimodal baseline.

### Performance under random split (scenario I)

We first evaluated SF-DTI under the random-split setting, which tests whether the model can reliably capture DTI patterns when training and test samples are drawn from similar distributions. As summarized in Tables 1 and 2, SF-DTI achieves competitive performance across the six benchmark datasets. Compared with the strongest competing method on each dataset, SF-DTI improves area under the receiver operating characteristic curve (AUROC)/area under the precision-recall curve (AUPRC) by 0.35%/0.61% on BindingDB, 3.39%/2.16% on BioSNAP, 5.15%/5.54% on DrugBank, and 1.08%/2.06% on KIBA, respectively. The clearer advantage on DrugBank may be related to the diversity of curated drug-target pairs, where jointly aligning molecular graph structure, pretrained chemical semantics, and PLM representations is beneficial. On KIBA, the imbalanced kinase-inhibitor benchmark makes AUPRC sensitive to interaction-aware representation quality, and the results suggest that SF-DTI benefits from multi-encoder protein fusion and cross-modal refinement. On Human and *C. elegans*, SF-DTI and MMDG-DTI obtain very close results, with each method leading one metric on each dataset, indicating comparable performance when strong baselines already achieve high AUROC and AUPRC values. The small standard deviations across repeated random partitions further suggest that the results are robust to data splitting.

**Table 1:** Performance comparison on BindingDB, BioSNAP, and DrugBank under scenario I

Method	BindingDB		BioSNAP		DrugBank	
	AUROC	AUPRC	AUROC	AUPRC	AUROC	AUPRC
TransformerCPI	0.8953 ± 0.0023	0.8601 ± 0.0030	0.8436 ± 0.0216	0.8590 ± 0.0157	0.8557 ± 0.0073	0.8584 ± 0.0077
MolTrans	0.9354 ± 0.0033	0.9084 ± 0.0044	0.8796 ± 0.0060	0.8829 ± 0.0071	0.8600 ± 0.0049	0.8360 ± 0.0020
Drug-BAN	0.9600 ± 0.0010	0.9480 ± 0.0020	0.9030 ± 0.0050	0.9020 ± 0.0040	0.8813 ± 0.0010	0.8740 ± 0.0020
BINDTI	0.9607 ± 0.0019	0.9446 ± 0.0024	0.9039 ± 0.0044	0.9020 ± 0.0065	0.8819 ± 0.0038	0.8830 ± 0.0044
MADTI	0.9660 ± 0.0010	0.9540 ± 0.0010	0.9040 ± 0.0020	0.9116 ± 0.0030	0.8706 ± 0.0030	0.8749 ± 0.0050
WDGBANDTI	0.9607 ± 0.0020	0.9491 ± 0.0030	0.9140 ± 0.0030	0.9250 ± 0.0020	0.8857 ± 0.0020	0.8848 ± 0.0030
MMDG-DTI	0.9567 ± 0.0035	0.9421 ± 0.0046	0.9127 ± 0.0020	0.9167 ± 0.0036	0.8922 ± 0.0020	0.8875 ± 0.0051
**SF-DTI**	**0.9695** ± **0.0020**	**0.9601** ± **0.0010**	**0.9479** ± **0.0010**	**0.9466** ± **0.0012**	**0.9437** ± **0.0010**	**0.9429** ± **0.0010**

The best value in each column is shown in bold.

**Table 2:** Performance comparison on Human, KIBA, and *C. elegans* under scenario I

Method	Human		KIBA		*C. elegans*	
	AUROC	AUPRC	AUROC	AUPRC	AUROC	AUPRC
TransformerCPI	0.9730 ± 0.0020	0.9790 ± 0.0020	0.9000 ± 0.0010	0.7850 ± 0.0020	0.9850 ± 0.0010	0.9850 ± 0.0020
MolTrans	0.9750 ± 0.0040	0.9770 ± 0.0030	0.9050 ± 0.0010	0.7880 ± 0.0030	0.9870 ± 0.0030	0.9890 ± 0.0020
Drug-BAN	0.9889 ± 0.0020	0.9912 ± 0.0020	0.9235 ± 0.0037	0.7820 ± 0.0113	0.9887 ± 0.0030	0.9903 ± 0.0020
BINDTI	0.9901 ± 0.0020	0.9910 ± 0.0020	0.9140 ± 0.0025	0.7884 ± 0.0010	0.9873 ± 0.0020	0.9895 ± 0.0010
MADTI	0.9880 ± 0.0020	0.9901 ± 0.0020	0.8922 ± 0.0060	0.7222 ± 0.0035	0.9840 ± 0.0025	0.9852 ± 0.0030
WDGBANDTI	0.9887 ± 0.0030	0.9915 ± 0.0020	0.9256 ± 0.0010	0.8093 ± 0.0020	0.9916 ± 0.0010	0.9925 ± 0.0014
MMDG-DTI	**0.9946** ± **0.0006**	0.9933 ± 0.0015	0.9353 ± 0.0035	0.8252 ± 0.0030	0.9958 ± 0.0010	**0.9965** ± **0.0009**
**SF-DTI**	0.9934 ± 0.0010	**0.9937** ± **0.0020**	**0.9461** ± **0.0013**	**0.8458** ± **0.0014**	**0.9960** ± **0.0009**	0.9963 ± 0.0010

The best value in each column is shown in bold.

### Performance under 5-fold cross-validation (scenario II)

Although the random-split setting provides an initial evaluation of predictive performance, the results may still be affected by how samples are partitioned into training and test sets. To obtain a more reliable and comprehensive assessment, we further evaluated SF-DTI under the 5-fold cross-validation setting. This scenario examines whether the performance advantage of SF-DTI remains stable across different data folds rather than being tied to a particular split. As reported in Tables 3 and 4, SF-DTI achieves the strongest overall performance under 5-fold cross-validation. The improvements remain clear on BindingDB, BioSNAP, DrugBank, and KIBA, while SF-DTI and MMDG-DTI obtain very close results on Human and *C. elegans*, where the leading methods already achieve high AUROC and AUPRC values. Overall, the performance was consistent across folds, indicating that the results were not driven by a single random partition.

**Table 3:** Performance comparison on BindingDB, BioSNAP, and DrugBank datasets under scenario II

Method	BindingDB		BioSNAP		DrugBank	
	AUROC	AUPRC	AUROC	AUPRC	AUROC	AUPRC
TransformerCPI	0.9409 ± 0.0033	0.9248 ± 0.0045	0.8966 ± 0.0066	0.9030 ± 0.0089	0.8718 ± 0.0044	0.8773 ± 0.0071
MolTrans	0.9418 ± 0.0049	0.9238 ± 0.0102	0.8895 ± 0.0072	0.8949 ± 0.0041	0.8709 ± 0.0080	0.8790 ± 0.0092
Drug-BAN	0.9650 ± 0.001	0.9530 ± 0.002	0.911 ± 0.004	0.908 ± 0.003	0.885 ± 0.003	0.879 ± 0.004
BINDTI	0.9655 ± 0.0036	0.9519 ± 0.0045	0.9046 ± 0.0028	0.9061 ± 0.0041	0.8881 ± 0.0066	0.8884 ± 0.0097
MADTI	0.9612 ± 0.0025	0.9513 ± 0.003	0.9102 ± 0.0016	0.901 ± 0.0028	0.882 ± 0.003	0.882 ± 0.004
WDGBANDTI	0.9643 ± 0.0015	0.9522 ± 0.0038	0.9117 ± 0.0032	0.9169 ± 0.0022	0.8856 ± 0.002	0.8892 ± 0.003
MMDG-DTI	0.9569 ± 0.0024	0.9450 ± 0.0037	0.9154 ± 0.0023	0.9218 ± 0.0027	0.8981 ± 0.0040	0.9002 ± 0.0033
**SF-DTI**	**0.9689** ± **0.0008**	**0.9563** ± **0.0028**	**0.9473** ± **0.0011**	**0.9468** ± **0.0017**	**0.9422** ± **0.0035**	**0.9403** ± **0.0027**

The best value in each column is shown in bold.

**Table 4:** Performance comparison on Human, KIBA, and *C. elegans* datasets under scenario II

Method	Human		KIBA		*C. elegans*	
	AUROC	AUPRC	AUROC	AUPRC	AUROC	AUPRC
TransformerCPI	0.9780 ± 0.0040	0.9801 ± 0.0030	0.9070 ± 0.0010	0.7648 ± 0.0020	0.9840 ± 0.0020	0.9830 ± 0.0030
MolTrans	0.9859 ± 0.0040	0.9802 ± 0.0030	0.9132 ± 0.0020	0.7949 ± 0.0016	0.9820 ± 0.0030	0.9820 ± 0.0030
Drug-BAN	0.9869 ± 0.0023	0.9889 ± 0.0035	0.9208 ± 0.0042	0.7833 ± 0.0101	0.9910 ± 0.0015	0.9895 ± 0.0043
BINDTI	0.9874 ± 0.0036	0.9853 ± 0.0030	0.9205 ± 0.0030	0.7992 ± 0.0044	0.9922 ± 0.0010	0.9913 ± 0.0020
MADTI	0.9887 ± 0.0012	0.9865 ± 0.0027	0.9199 ± 0.0020	0.7883 ± 0.0050	0.9886 ± 0.0020	0.9875 ± 0.0030
WDGBANDTI	0.9866 ± 0.0017	0.9876 ± 0.0022	0.9258 ± 0.0018	0.7949 ± 0.0054	0.9914 ± 0.0027	0.9913 ± 0.0033
MMDG-DTI	0.9908 ± 0.0030	**0.9927** ± **0.0026**	0.9304 ± 0.0051	0.8123 ± 0.0104	0.9957 ± 0.0009	0.9961 ± 0.0009
**SF-DTI**	**0.9912** ± **0.0017**	0.9916 ± 0.0015	**0.9432** ± **0.0021**	**0.8393** ± **0.0032**	**0.9962** ± **0.0009**	**0.9964** ± **0.0008**

The best value in each column is shown in bold.

In addition, we performed two-sided paired *t* tests between SF-DTI and the baseline methods under random split and 5-fold cross-validation. The tests were based on results from five random seeds for random split and 5-folds for cross-validation. To control the family-wise error rate arising from multiple baseline comparisons, the *p* values were adjusted using the Holm-Bonferroni procedure separately for each dataset, evaluation metric, and evaluation protocol. A Holm-adjusted *p* value below 0.05 was considered statistically significant. As shown in Tables S1 and S2, the adjusted tests indicated statistically significant differences in favor of SF-DTI against each evaluated baseline on BioSNAP and DrugBank for both metrics under both evaluation protocols. On KIBA, the differences remained statistically significant except for the random-split AUPRC comparison with MMDG-DTI. On BindingDB, statistical significance was observed in a subset of comparisons and varied across metrics and evaluation protocols. On Human and *C. elegans*, no statistically significant difference was observed between SF-DTI and MMDG-DTI. These results provide statistical support for the performance differences on several benchmarks while also identifying comparisons in which the evaluated methods achieved comparable performance.

### Performance under unseen-drug and unseen-target splits (scenario III)

To evaluate cold-start generalization, we assessed SF-DTI on BindingDB, BioSNAP, and DrugBank under unseen-drug and unseen-target settings. In the unseen-drug setting, all interactions involving the held-out test drugs were excluded from training; analogously, in the unseen-target setting, all interactions involving the held-out test proteins were excluded from training. The model was, therefore, required to predict interactions involving entities not observed during training. SF-DTI achieved the highest AUROC among the external comparison methods in all six settings and the highest AUPRC in five of the six settings (Table 5).

**Table 5:** Performance comparison under unseen-drug and unseen-target settings (scenario III)

Method	AUROC					
	BindingDB		BioSNAP		DrugBank	
	Unseen drug	Unseen target	Unseen drug	Unseen target	Unseen drug	Unseen target
TransformerCPI	0.9050 ± 0.0080	0.5664 ± 0.0137	0.8580 ± 0.0050	0.7620 ± 0.0300	0.7566 ± 0.0357	0.6483 ± 0.0077
MolTrans	0.9182 ± 0.0014	0.5500 ± 0.1720	0.8530 ± 0.0110	0.7700 ± 0.0290	0.7702 ± 0.0087	0.6845 ± 0.0112
Drug-BAN	0.9492 ± 0.0012	0.6263 ± 0.0108	0.8620 ± 0.0020	0.7710 ± 0.0240	0.8286 ± 0.0053	0.6673 ± 0.0133
BINDTI	0.9468 ± 0.0020	0.5869 ± 0.0370	0.8650 ± 0.0011	0.6169 ± 0.0421	0.8150 ± 0.0036	0.6708 ± 0.0245
MADTI	0.9484 ± 0.0033	0.5900 ± 0.0171	0.8720 ± 0.0040	0.7810 ± 0.0240	0.8098 ± 0.0069	0.7174 ± 0.0143
WDGBANDTI	0.9505 ± 0.0017	0.5898 ± 0.0362	0.8513 ± 0.0020	0.6320 ± 0.0020	0.8186 ± 0.0056	0.6940 ± 0.0106
MMDG-DTI	0.9427 ± 0.0027	0.6351 ± 0.0073	0.8722 ± 0.0084	0.7901 ± 0.0177	0.8096 ± 0.0035	0.8013 ± 0.0092
Concat-MLP^a^	0.9412 ± 0.0018	0.6514 ± 0.0070	0.8782 ± 0.0017	0.8657 ± 0.0059	0.8602 ± 0.0010	0.8639 ± 0.0017
**SF-DTI**	**0.9550** ± **0.0025**	**0.6770** ± **0.0151**	**0.9051** ± **0.0021**	**0.8807** ± **0.0025**	**0.8919** ± **0.0080**	**0.8810** ± **0.0037**

Method	AUPRC					
	BindingDB		BioSNAP		DrugBank	
	Unseen drug	Unseen target	Unseen drug	Unseen target	Unseen drug	Unseen target
TransformerCPI	0.7741 ± 0.0042	0.5008 ± 0.0443	0.8134 ± 0.0269	0.5946 ± 0.0490	0.7801 ± 0.0223	0.6684 ± 0.0014
MolTrans	0.8816 ± 0.0045	0.5117 ± 0.0213	0.8663 ± 0.0010	0.6521 ± 0.0305	0.7898 ± 0.0047	0.7121 ± 0.0032
Drug-BAN	0.9324 ± 0.0007	**0.5770** ± **0.0205**	0.8857 ± 0.0021	0.6339 ± 0.0250	0.8383 ± 0.0042	0.6894 ± 0.0122
BINDTI	0.9306 ± 0.0030	0.5090 ± 0.0274	0.8848 ± 0.0038	0.6381 ± 0.0252	0.8258 ± 0.0035	0.6867 ± 0.0254
MADTI	0.9296 ± 0.0050	0.5092 ± 0.0213	0.8876 ± 0.0050	0.7133 ± 0.0234	0.8164 ± 0.0057	0.7335 ± 0.0164
WDGBANDTI	0.9352 ± 0.0035	0.5225 ± 0.0420	0.8821 ± 0.0049	0.6504 ± 0.0239	0.8303 ± 0.0037	0.7087 ± 0.0116
MMDG-DTI	0.9255 ± 0.0049	0.5370 ± 0.0207	0.8930 ± 0.0053	0.8357 ± 0.0114	0.8412 ± 0.0044	0.8414 ± 0.0114
Concat-MLP^a^	0.9206 ± 0.0031	0.5207 ± 0.0133	0.8946 ± 0.0015	0.8643 ± 0.0061	0.8647 ± 0.0018	0.8686 ± 0.0005
**SF-DTI**	**0.9406** ± **0.0032**	0.5572 ± 0.0226	**0.9155** ± **0.0060**	**0.8837** ± **0.0034**	**0.8947** ± **0.0076**	**0.8884** ± **0.0042**

Values are reported as mean ± standard deviation. The best value in each column is shown in bold.

a Concat-MLP uses the same pre-extracted ChemBERTa, ESM-2, and ProtT5 representations as SF-DTI, with the proposed fusion architecture replaced by direct concatenation and an MLP classifier.

To assess whether the cold-start gains were attributable solely to the pretrained representations, we evaluated a concatention-based multilayer perceptron (Concat-MLP) baseline using the same pre-extracted ChemBERTa, ESM-2, and ProtT5 features used by SF-DTI. SF-DTI consistently outperformed this pretrained-feature control across all six settings, with AUROC improvements of 1.38%–3.17% and AUPRC improvements of 1.94%–3.65%. These gains were observed for both unseen-drug and unseen-target prediction across all three datasets, indicating that the cold-start performance was not attributable solely to the pretrained representations and that the full SF-DTI architecture provided additional benefits when predicting interactions involving previously unseen entities.

Relative to the strongest external method, the improvement was modest on BindingDB under the unseen-drug setting, reaching 0.45% in AUROC and 0.54% in AUPRC, where several methods already approached high performance. Larger gains were observed on BioSNAP, where SF-DTI improved AUROC/AUPRC by 3.29%/2.25% under unseen-drug prediction and by 9.06%/4.80% under unseen-target prediction. On DrugBank, the corresponding improvements were 6.33%/5.35% and 7.97%/4.70%, respectively. These results show that the advantage of SF-DTI was particularly pronounced in settings involving larger entity-level distribution shifts.

BindingDB unseen-target prediction remained the most challenging condition, yielding the lowest AUROC and AUPRC for SF-DTI among the six settings. SF-DTI achieved the highest AUROC of 0.6770, outperforming the strongest external method by 4.19% and the same-feature Concat-MLP control by 2.56%. However, Drug-BAN achieved an AUPRC of 0.5770, which was 1.98% higher than that of SF-DTI. The lower absolute performance and the metric-dependent ranking indicate that, although SF-DTI improved overall ranking discrimination, reliable retrieval of positive interactions involving previously unseen BindingDB proteins remains difficult.

### Performance under cluster split (scenario IV)

To evaluate SF-DTI under distribution shift, we adopted the clustering-based pair split used in DrugBAN.^28^ This protocol induces an entity-level distribution shift between the source and target domains and is, therefore, more challenging than standard random-split evaluation. The standard SF-DTI model was trained using labeled source-domain pairs and evaluated on target-domain test pairs without an additional domain-adaptation objective.

As shown in Figure 1, the evaluated methods showed different advantages across metrics: SF-DTI improved AUROC by 1.65% over MADTI_CADA_, whereas MADTI_CADA_ retained a 3.71% advantage in AUPRC. On BioSNAP, SF-DTI improved AUROC/AUPRC by 2.37%/0.70% over MMDG-DTI. Applying a conditional domain advasarial network (CDAN) to SF-DTI provided consistent additional gains, increasing AUROC/AUPRC by 0.67%/1.58% on BindingDB and by 1.15%/1.26% on BioSNAP.

**Figure 1:**
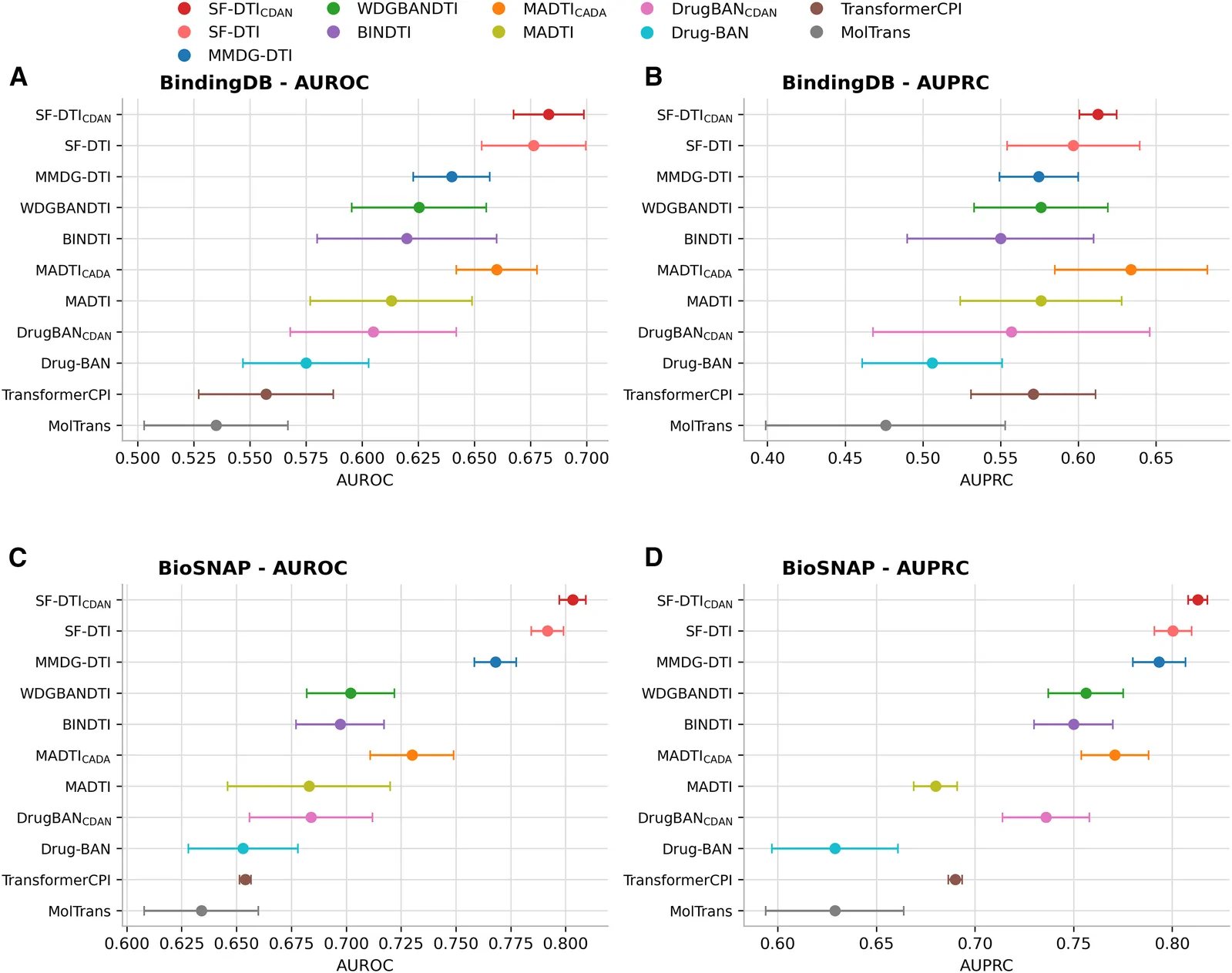
Performance comparison under the clustering-based pair split (scenario IV) on BindingDB and BioSNAP Panels show (A) AUROC on BindingDB, (B) AUPRC on BindingDB, (C) AUROC on BioSNAP, and (D) AUPRC on BioSNAP. Labels without subscripts denote standard clustering-based models, whereas CDAN and CADA subscripts denote the corresponding domain-adaptation variants. Different colors denote different methods. Points represent mean AUROC and AUPRC values, and error bars represent standard deviations.

In this implementation, CDAN aligns the pooled interaction representations using target-domain pairs without access to their interaction labels. The comparison between SF-DTI and SF-DTI_CDAN_ therefore isolates the incremental contribution of representation-level domain alignment under the evaluated protocol. The consistent but moderate improvements suggest that the standard SF-DTI architecture already retains substantial predictive capacity under the cluster-based distribution shift, while domain alignment provides an additional benefit.

Beyond aggregate performance, we further examined the remaining prediction errors under the cold-start and cross-domain settings. In scenario III, the difference between unseen-drug and unseen-target prediction was most pronounced on BindingDB, where AUROC decreased from 0.9550 under the unseen-drug setting to 0.6770 under the unseen-target setting. This result indicates that generalization to previously unseen proteins is substantially more challenging than generalization to unseen drugs on this dataset. In scenario IV, representative false-positive and false-negative predictions produced by the standard SF-DTI model on the BioSNAP and BindingDB target-domain test sets are reported in Tables S15 and S16, respectively. Relative to the benchmark labels, the false-positive cases received high predicted probabilities despite having negative labels, whereas the false-negative cases received low probabilities despite having positive labels. The BioSNAP examples included a small number of highly confident errors despite the comparatively stronger aggregate performance on this dataset. In contrast, the selected BindingDB errors were less extreme, but the lower aggregate AUROC and AUPRC were consistent with a broader loss of ranking ability under cluster-defined distribution shift. These complementary failure patterns highlight residual target-domain prediction difficulties that are not fully reflected by aggregate evaluation metrics alone.

### Ablation study

To evaluate the contribution of each component in SF-DTI, we conducted a series of ablation studies. We first removed the three major modules, namely the MPMI module, the SCMAF module, and the cascaded CMSPF module, to assess their individual effects on prediction performance. We then varied the cascade depth to examine whether multiple rounds of cross-modal refinement are beneficial. The main component ablation results are shown in Table 6, with complete mean and standard deviation values provided in Table S4. The cascade-depth results are shown in Table 7, with the corresponding statistics provided in Table S8. In addition, to distinguish the contribution of the proposed fusion architecture from the effect of pretrained representations alone, we included pretrained-representation controls and single-/dual-encoder protein controls in Tables S5 and S6. Fine-grained ablations of MPMI, SCMAF, and CMSPF are further provided in Table S7.

**Table 6:** Ablation experiments on the main modules of SF-DTI

Method variant	BindingDB		BioSNAP		DrugBank		Human		KIBA		*C. elegans*	
	AUC	AUPR	AUC	AUPR	AUC	AUPR	AUC	AUPR	AUC	AUPR	AUC	AUPR
**SF-DTI (full)**	**0.9695**	**0.9601**	**0.9479**	**0.9466**	**0.9437**	**0.9429**	**0.9934**	**0.9937**	**0.9461**	**0.8458**	**0.9960**	**0.9963**
w/o MPMI	0.9642	0.9465	0.9413	0.9318	0.9358	0.9329	0.9916	0.9931	0.9397	0.8323	0.9931	0.9939
w/o SCMAF	0.9633	0.9503	0.9427	0.9423	0.9408	0.9357	0.9895	0.9907	0.9417	0.8366	0.9915	0.9891
w/o CMSPF	0.9652	0.9475	0.9429	0.9350	0.9373	0.9324	0.9930	0.9936	0.9409	0.8347	0.9947	0.9930

The best value in each column is shown in bold.

**Table 7:** Impact of cascade depth in CMSPF module

Method variant	BindingDB		BioSNAP		DrugBank		Human		KIBA		*C. elegans*	
	AUC	AUPR	AUC	AUPR	AUC	AUPR	AUC	AUPR	AUC	AUPR	AUC	AUPR
1 cascade	0.9668	0.9522	0.9421	0.9368	0.9402	0.9343	0.9931	0.9936	0.9392	0.8251	0.9954	0.9945
2 cascades	0.9664	0.9517	0.9443	0.9404	0.9424	0.9374	0.9919	0.9927	0.9408	0.8269	0.9949	0.9950
3 cascades	0.9647	0.9496	0.9436	0.9389	0.9411	0.9336	0.9927	0.9936	0.9382	0.8232	0.9943	0.9943
**4 cascades (default)**	**0.9695**	**0.9601**	**0.9479**	**0.9466**	**0.9437**	**0.9429**	**0.9934**	**0.9937**	**0.9461**	**0.8458**	**0.9960**	**0.9963**

The best value in each column is shown in bold.

To validate the effectiveness of each major component, we studied three ablated variants of SF-DTI: one without MPMI, one without SCMAF, and one without CMSPF. For the SCMAF variant, the proposed alignment-based drug fusion module was replaced by a simple concatenation-based fusion strategy. As shown in Table 6, removing any of the three modules led to performance degradation across most datasets, indicating that all of them contribute to the final performance of SF-DTI.

The first variant, without MPMI, examines the role of the protein fusion module. Compared with the full model, removing MPMI resulted in consistent drops on all six datasets, with particularly clear declines on BindingDB and BioSNAP. For example, the AUPRC decreased from 0.9601 to 0.9465 on BindingDB and from 0.9466 to 0.9318 on BioSNAP. These results suggest that integrating complementary protein representations from multiple pretrained encoders is beneficial for robust DTI prediction.

The second variant, without SCMAF, evaluates the effect of the proposed drug structure-semantic fusion strategy. When SCMAF was replaced by simple concatenation, the performance again decreased across most datasets. The drop was particularly evident in AUPRC, for example, from 0.9601 to 0.9503 on BindingDB and from 0.8458 to 0.8366 on KIBA. This result suggests that directly concatenating graph-derived molecular structural features and pretrained chemical semantic embeddings is insufficient for effective fusion, and that explicit alignment is important for reducing representation inconsistency between the two drug-side modalities.

The third variant, without CMSPF, evaluates the contribution of the cascaded cross-modal refinement mechanism. Removing CMSPF also caused clear degradation across the datasets, especially on BindingDB, BioSNAP, DrugBank, and KIBA. For example, the AUPRC dropped from 0.9601 to 0.9475 on BindingDB, from 0.9466 to 0.9350 on BioSNAP, and from 0.8458 to 0.8347 on KIBA. These results indicate that progressive cross-modal signal propagation plays an important role in refining interaction-aware representations.

To distinguish the contribution of the proposed fusion architecture from that of pretrained representations, we evaluated Concat-MLP, bilinear fusion, cross-attention, and gated-fusion controls using the same frozen, pooled ChemBERTa, ESM-2, and ProtT5 features, as reported in Table S5. These controls excluded the GCN-derived molecular graph representation and, therefore, assessed the benefit of the proposed architecture over simpler fusion of the same pretrained representations. The contribution of graph-derived molecular features was evaluated separately through the component-level and fine-grained SCMAF ablations. Compared with Concat-MLP, SF-DTI improved the AUPRC from 0.9372 to 0.9601 on BindingDB, from 0.9255 to 0.9466 on BioSNAP, from 0.9193 to 0.9429 on DrugBank, and from 0.7700 to 0.8458 on KIBA. SF-DTI also achieved higher AUPRC than the bilinear, cross-attention, and gated-fusion controls on these representative datasets. Together, these complementary controls indicate that the performance of SF-DTI depends on both heterogeneous representation integration and the proposed alignment and cross-modal refinement mechanisms.

We further evaluated the contribution of protein-side pretrained representations using single-/dual-encoder protein controls, as reported in Table S6. Under the same Concat-MLP downstream architecture and a fixed ChemBERTa drug representation, the composition of the pre-extracted protein representations was varied among ESM-2, ProtT5, and ESM-2+ProtT5, showing dataset-dependent performance patterns. For example, the dual-encoder setting achieved the best AUPRC on BindingDB and KIBA, whereas ProtT5 alone performed better on several other datasets. This suggests that ESM-2 and ProtT5 provide complementary but not identical protein information. At the same time, simple concatenation of the two pretrained protein representations does not consistently yield the best result across all datasets, suggesting that explicit protein-side interaction modeling can provide benefits beyond simple feature concatenation.

To further examine the contributions of the individual mechanisms within the proposed modules, we conducted fine-grained ablations of MPMI, SCMAF, and CMSPF, as reported in Table S7. For MPMI, removing the bidirectional interaction or reducing the module to projection-only fusion weakened performance, particularly on KIBA, where the AUPRC decreased from 0.8458 to 0.8334 and 0.8265, respectively. For SCMAF, removing the shared subspace constraint, replacing the alignment module with average fusion, or using projection-only fusion also reduced performance, indicating that explicit structure-semantic alignment is more effective than simple averaging or shallow projection. For CMSPF, replacing the energy-guided routing with fixed-gate or learned-scalar-gate variants generally led to lower AUPRC on BioSNAP, DrugBank, and KIBA. These fine-grained results further support that the improvement of SF-DTI comes not only from the use of pretrained encoders but also from the specific architectural mechanisms designed for protein mutual interaction, drug structure-semantic alignment, and progressive cross-modal signal routing.

To examine the effect of cascade depth, we further studied four configurations with 1, 2, 3, and 4 cascades. The results are reported in Table 7, with the corresponding statistics provided in Table S8. In general, increasing the cascade depth improved the performance of the model, although minor fluctuations were still observed at intermediate depths. The 4-cascade configuration achieved the best overall results across the six datasets, suggesting that multiple rounds of cross-modal refinement are beneficial for interaction modeling. Considering both performance and model complexity, we used 4 cascades as the default setting in the final model. Hyperparameter sensitivity on DrugBank was further evaluated with repeated runs for cascade depth, attention heads, fusion dimension, residual weight, and learning rate, as reported in Table S9.

### Visualization of learned manifolds

To investigate whether SF-DTI learns a well-structured cross-modal representation space, we visualize the fused features using uniform manifold approximation and projection (UMAP).^36^ Figure 2 presents the two-dimensional projections of randomly sampled test pairs (1,200 pairs per dataset) from BioSNAP, DrugBank, and Human. From the modality view, drug and protein features remain distinguishable while being organized within a shared representation space, indicating that the model preserves modality-specific characteristics while enabling effective cross-modal fusion. From the label view, positive and negative pairs exhibit a noticeable degree of separation, and the predicted-label view largely agrees with the ground-truth distribution. Overall, these visualizations suggest that SF-DTI learns a structured and discriminative feature manifold in which biologically meaningful cross-modal interaction patterns become more distinguishable. For completeness, the full projection files, plotting code, and the ranked score lists used in the case studies are publicly available at https://github.com/wbw176-netizen/SF-DTI/tree/main/visualization.

**Figure 2:**
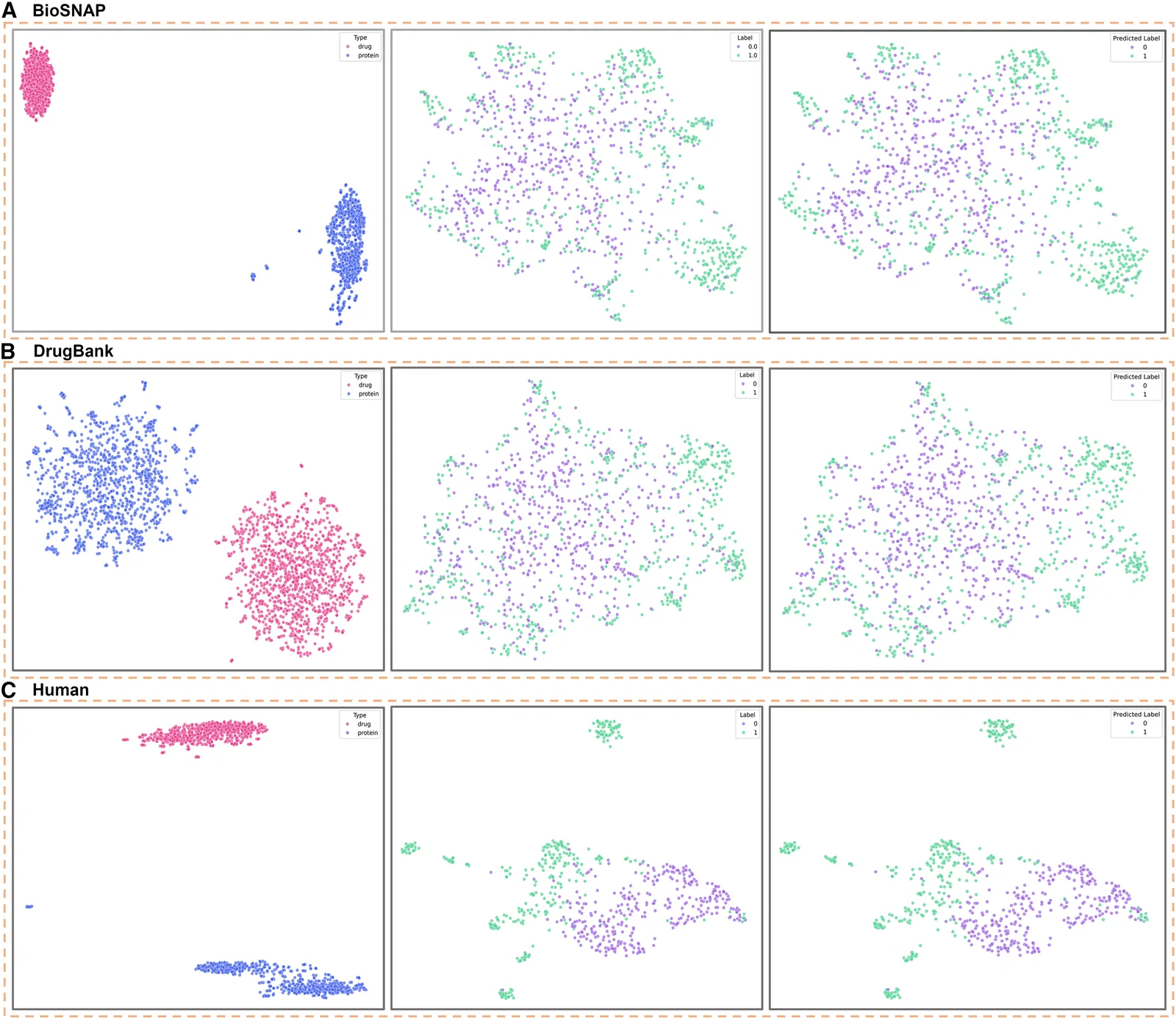
UMAP visualization of learned representations on the BioSNAP, DrugBank, and Human datasets The three rows correspond to (A) BioSNAP, (B) DrugBank, and (C) Human, respectively, and each column shows a different view of the learned feature space. Column 1 visualizes modality types, where drug features are shown in magenta and protein features in indigo. Column 2 shows the distributions of samples colored by ground-truth interaction labels, where purple denotes non-interacting pairs and green denotes interacting pairs. Column 3 shows the same samples colored by predicted labels, illustrating the consistency between the learned representations and the final predictions.

### Case studies

Although SF-DTI demonstrated strong DTI classification performance across the benchmark datasets, these results alone do not fully demonstrate whether it can effectively prioritize unobserved candidate DTIs. To further evaluate this capability, we conducted blinded case studies from both drug-centric and target-centric perspectives. For each query, a query-level blinded split was constructed from deduplicated DrugBank pairs. Duplicate entries with the same DrugBank ID and UniProt ID were collapsed, and the positive interaction label was retained when duplicate records had conflicting labels. In the drug-centric setting, all pairs involving the query drug were excluded from both the training and validation sets; analogously, in the target-centric setting, all pairs involving the query target were excluded. The excluded query-associated pairs, comprising DrugBank-annotated positive interactions and candidate pairs without confirmed interaction annotations, constituted the blinded candidate pool for ranking. The model was trained and selected using only the remaining pairs. After model development was complete, all pairs in the corresponding blinded candidate pool were ranked according to their predicted interaction probabilities. The top-ranked predictions were then validated against the DrugBank annotations used in this study.^37^ The number of pairs in each blinded candidate pool and the number of DrugBank-annotated positive pairs are reported in Table S10.

For the drug-centric analysis, we selected three DrugBank compounds, namely NADH (DrugBank: DB00157), beta-D-glucose (DrugBank: DB02379), and flavin adenine dinucleotide (FAD; DrugBank: DB03147).^37^ NADH and FAD are important cofactors involved in cellular metabolism and redox processes.^38^^,^^39^ As summarized in Tables 8 and 9, the top-10 predictions for all three query drugs corresponded to DrugBank-supported interactions in their respective blinded candidate pools. These results indicate that SF-DTI can effectively identify DTIs after all training and validation pairs involving each query drug have been removed.

**Table 8:** Top-ranked protein targets predicted for NADH and beta-D-glucose

Rank	NADH (DrugBank: DB00157)		Beta-D-glucose (DrugBank: DB02379)	
	UniProt ID	Result	UniProt ID	Result
1	P11586	confirmed	P19961	confirmed
2	P04406	confirmed	P04745	confirmed
3	P48728	confirmed	P04746	confirmed
4	P16219	confirmed	P01556	confirmed
5	P21695	confirmed	P12528	confirmed
6	P20839	confirmed	Q9ZEU2	confirmed
7	P12268	confirmed	P07986	confirmed
8	Q99714	confirmed	P54583	confirmed
9	P40926	confirmed	P40943	confirmed
10	Q9UDR5	confirmed	Q9KG76	confirmed

Scores are omitted for compact presentation. All listed pairs correspond to DrugBank-supported interactions in the blinded candidate pools.

**Table 10:** Top-ranked drug candidates predicted to interact with H1R and ADRA1C

Rank	H1R (UniProt: P35367)		ADRA1C (UniProt: P35348)	
	DrugBank ID	Result	DrugBank ID	Result
1	DB00334	confirmed	DB01624	confirmed
2	DB00363	confirmed	DB01136	confirmed
3	DB01624	confirmed	DB13025	confirmed
4	DB00246	confirmed	DB00243	confirmed
5	DB00321	confirmed	DB01238	confirmed
6	DB00751	confirmed	DB04855	confirmed
7	DB00408	confirmed	DB09167	confirmed
8	DB00543	confirmed	DB00334	confirmed
9	DB00540	confirmed	DB04946	confirmed
10	DB01151	confirmed	DB00363	confirmed

Scores are omitted for compact presentation. All listed pairs correspond to DrugBank-supported interactions in the blinded candidate pools.

For the target-centric analysis, we focused on H1R (UniProt: P35367), ADRA1C (UniProt: P35348), ADRA2A (UniProt: P08913), BACE1 (UniProt: P56817), and HTR7 (UniProt: P34969).^40^^,^^41^^,^^42^^,^^43^^,^^44^ Compared with the initial receptor-focused cases, BACE1 and HTR7 further test whether SF-DTI maintains top-ranked retrieval on targets with fewer retained candidate pairs. As shown in Tables 9 and 10, SF-DTI ranked DrugBank-supported interactions among the top candidates for H1R, ADRA1C, and ADRA2A after all training and validation pairs involving the corresponding query target had been removed. The additional ranked lists for BACE1 and HTR7 are provided in Table S11. HTR7 yielded 10 DrugBank-confirmed interactions among the top 10 candidates, whereas BACE1 yielded 8, further supporting the ability of SF-DTI to prioritize relevant drug candidates under target-level blinding.

**Table 9:** Top-ranked drug candidates predicted to interact with ADRA2A (UniProt: P08913) and top-ranked protein targets predicted for FAD (DrugBank: DB03147)

Rank	ADRA2A (UniProt: P08913)		FAD (DrugBank: DB03147)	
	DrugBank ID	Result	UniProt ID	Result
1	DB01136	confirmed	Q16881	confirmed
2	DB00715	confirmed	P37062	confirmed
3	DB09286	confirmed	P16640	confirmed
4	DB01392	confirmed	P14920	confirmed
5	DB13345	confirmed	P22570	confirmed
6	DB01624	confirmed	Q92947	confirmed
7	DB00320	confirmed	Q52437	confirmed
8	DB09202	confirmed	P16219	confirmed
9	DB04855	confirmed	Q9UKU7	confirmed
10	DB11273	confirmed	Q9LAG2	confirmed

Scores are omitted for compact presentation. All listed pairs correspond to DrugBank-supported interactions in the blinded candidate pools.

Ranked retrieval metrics are summarized in Table 11. Hit@k was calculated as the number of DrugBank-supported interactions among the top-k predictions in the corresponding blinded candidate pool. Recall@10 denotes the fraction of all DrugBank-supported interactions associated with a query that were retrieved among the top-10 candidates, and EF@10 denotes the top-10 hit rate divided by the corresponding candidate-pool interaction rate. SF-DTI achieved Hit@1 of 8/8, Hit@5 of 39/40, and Hit@10 of 78/80 across the eight blinded queries, with an overall Recall@10 of 0.112 and mean EF@10 of 1.063. Across the eight blinded queries, EF@10 ranged from 1.000 to 1.297, with the highest enrichment observed for HTR7 (1.297), followed by BACE1 (1.126). Together with the aggregate Hit@1, Hit@5, and Hit@10 results, these findings demonstrate that SF-DTI effectively prioritized DrugBank-supported interactions across the selected drug-centric and target-centric queries.

**Table 11:** Quantitative retrieval metrics in the blinded case studies

Query	Setting	Hit@1	Hit@5	Hit@10	Recall@10	EF@10
NADH	drug-centric	1/1	5/5	10/10	0.069	1.007
Beta-D-glucose	drug-centric	1/1	5/5	10/10	0.111	1.022
FAD	drug-centric	1/1	5/5	10/10	0.116	1.023
H1R	target-centric	1/1	5/5	10/10	0.099	1.000
ADRA1C	target-centric	1/1	5/5	10/10	0.087	1.026
ADRA2A	target-centric	1/1	5/5	10/10	0.105	1.000
BACE1	target-centric	1/1	4/5	8/10	0.296	1.126
HTR7	target-centric	1/1	5/5	10/10	0.270	1.297
Overall	–	8/8	39/40	78/80	0.112	1.063

### Activation-based interpretability analysis

A further strength of SF-DTI is to enable molecular-level interpretation by visualizing the contribution of ligand substructures to the final predictive result. Here, we examined six co-crystallized ligands from the Protein Data Bank (PDB),^45^ including PDB: 1B6K,^46^ 1IEP,^47^ 1X70,^48^ 3EIG,^49^ 5W8L,^50^ and 6QL2.^51^ The corresponding PDB complexes and resolutions are listed in Table S12. Only X-ray structures with resolution better than 2.5 Å were considered, and the corresponding drug-target pairs were not part of the training set. The visualization results are shown in Figure 3 alongside the ligand-protein interaction maps originating from the corresponding X-ray structures. For each molecule, the top 20% activation-weighted atoms were colored in orange.

**Figure 3:**
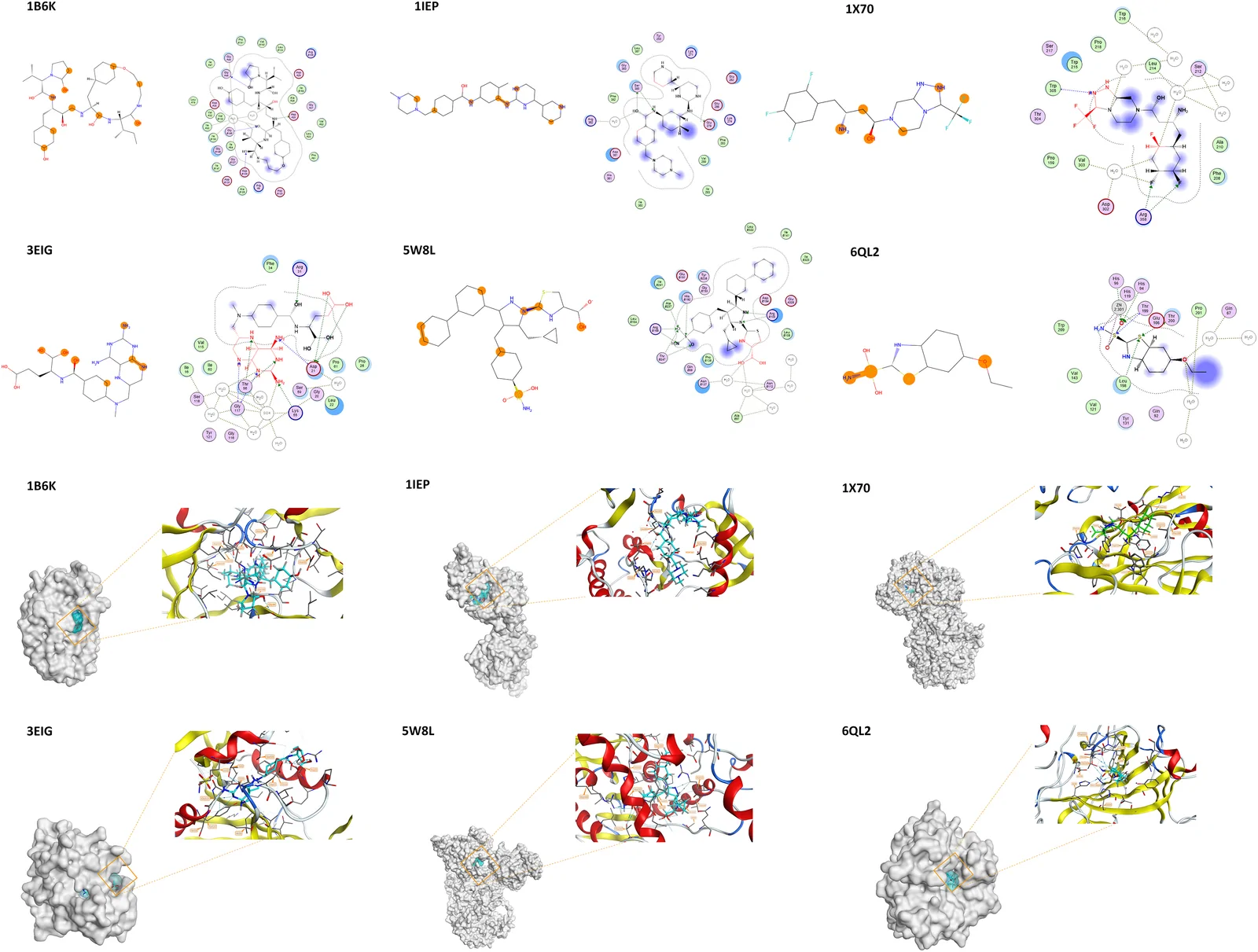
Importance visualization of ligands and binding pockets The two-dimensional ligand structures show highlighted atoms (orange) predicted to contribute to protein binding. The corresponding ligand-protein interaction maps and three-dimensional binding-pocket views from the X-ray structures are provided for comparison. Ligand structures were visualized using RDKit,^52^ and ligand-protein interaction maps and three-dimensional views were rendered using Molecular Operating Environment (MOE).^53^

For the complex of ethoxzolamide with human carbonic anhydrase II (PDB: 6QL2), SF-DTI concentrated high-weight atoms around the sulfonamide-containing region, a key interaction group in the crystal structure. The highlighted sulfonamide nitrogen was located close to Thr199, and the highlighted sulfur and oxygen atoms fell within the same polar pocket shown in the interaction map. Meanwhile, not every highlighted atom formed an individual contact under the distance cutoff. Therefore, the activation map should be read as local substructure importance rather than a one-to-one physical contact annotation.

In the crystal structure of human L-lactate dehydrogenase A in complex with the 9YA ligand (PDB: 5W8L), the activation visualization again highlighted ligand regions associated with the binding pocket. The highlighted O38 atom was close to Thr247, while N16 and C31 were located near Asn137. Several highlighted carbon atoms also appeared on the neighboring aromatic scaffold, consistent with the interaction map in which aromatic groups participate in the local binding environment. Compared with ethoxzolamide, 9YA has a larger and more complex scaffold, yet SF-DTI still emphasized multiple pocket-facing moieties rather than distributing high weights uniformly over the molecule.

In the structure of the PI5 ligand bound to HIV-1 protease (PDB: 1B6K), highlighted oxygen and nitrogen atoms were located near catalytic-pocket residues, including Asp25, Asp29, Gly27, and Asp129, while several highlighted carbon atoms were distributed on the macrocyclic scaffold. In thestructure of the STI-571/imatinib ligand bound to ABL kinase (PDB: 1IEP), multiple high-weight nitrogen atoms were positioned near Met318, Thr315, Glu286, and Phe382, and the terminal highlighted carbon was close to Asp381. In the structure of the 715 ligand bound to dipeptidyl peptidase IV (PDB: 1X70), high-weight atoms appeared near Glu205, Glu206, and Ser209, although two highlighted atoms near Phe357 did not satisfy the contact cutoff. In the structure of the methotrexate ligand bound to dihydrofolate reductase (PDB: 3EIG), the highlighted methotrexate atoms included polar atoms near Glu30, Asn64, and Arg70, with the carboxylate oxygen atoms showing clear correspondence to the Arg70 region. These examples indicate that ligand-side activations tend to mark chemically meaningful functional groups and neighboring scaffold regions involved in local recognition, while also exposing imperfect atom-level assignments in some cases.

As for the more challenging protein-side interpretability, the results were less direct than those for the ligand-side visualization. This is expected because SF-DTI uses sequence-level protein representations, and 1D protein sequences do not necessarily imply the 3D configuration and locality of the binding pocket. Therefore, highlighted protein regions should not be interpreted as exact residue-level physical contacts. Nevertheless, the repeated agreement between ligand-side activation regions and experimentally observed binding environments suggests that SF-DTI can identify chemically plausible substructures relevant to drug-target recognition.

## Discussion

In this work, we present SF-DTI, a unified deep learning framework for DTI prediction. The overall SF-DTI architecture and the pre-extracted feature pipeline are illustrated in Figures 4 and 5, respectively. By integrating multi-encoder protein fusion, subspace-constrained drug representation alignment, and cascaded cross-modal refinement, SF-DTI enhances cross-modal interaction modeling and achieves competitive predictive performance across standard, cold-start, and cross-domain settings. Beyond the quantitative comparisons, ablation studies, manifold visualizations, blinded candidate-prioritization case studies, and interpretability analyses collectively suggest that the model learns interaction-relevant and biologically plausible patterns. More broadly, the proposed CMSPF mechanism may inform future efforts to model interactions between heterogeneous representations in biomedical multimodal learning.

**Figure 4:**
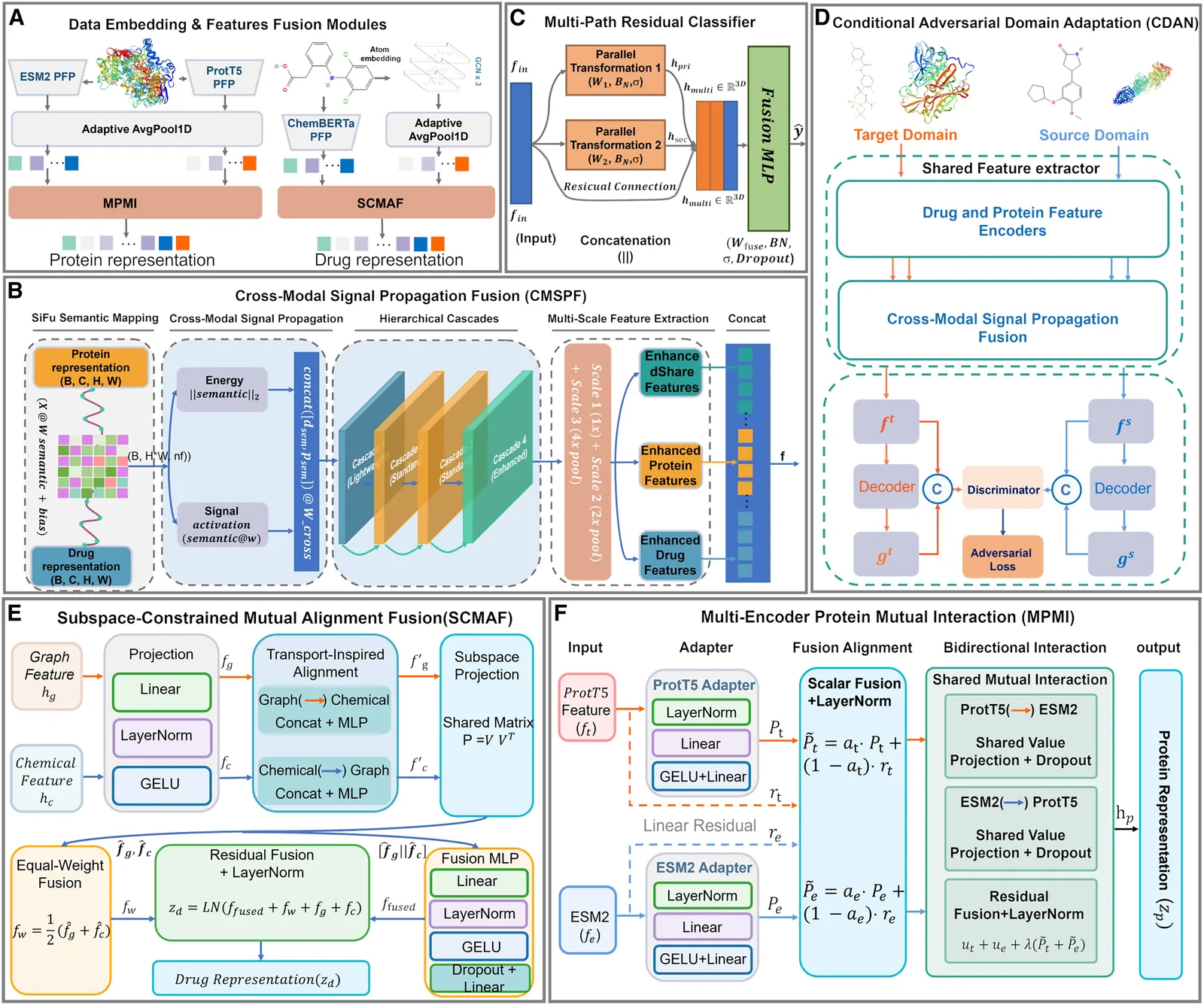
Overview of the SF-DTI framework (A) Data embedding and fusion encode proteins and drugs and produce fused features. (B) Cascaded cross-modal signal propagation fusion (CMSPF) that iteratively aligns and routes signals among global modality representations. (C) Multi-Path Residual Classifier (MPRC) that processes the input features through parallel pathways with a residual connection. (D) CDAN-based auxiliary domain adaptation used in scenario IV. (E) Subspace-constrained mutual alignment fusion (SCMAF) module for aligning and integrating graph-based structural features with pretrained molecular embeddings. (F) Multi-encoder protein mutual interaction (MPMI) module for integrating complementary global protein representations from multiple pretrained protein language models.

**Figure 5:**
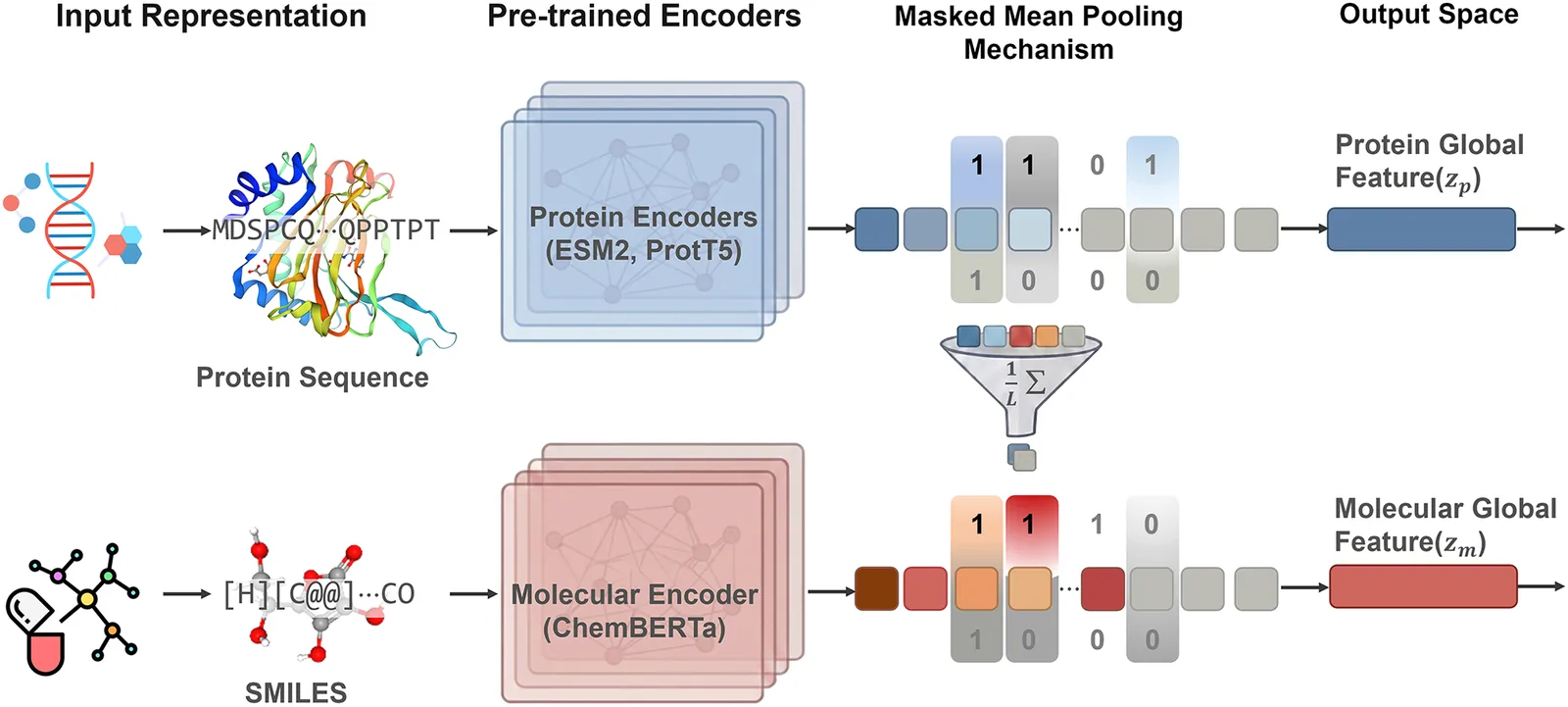
Overview of the pre-extracted feature pipeline Raw sequence inputs, including protein sequences and molecular Simplified Molecular Input Line Entry System (SMILES), are processed by frozen pretrained encoders (ESM-2 and ProtT5 for proteins, and ChemBERTa for molecules). A masked mean pooling mechanism then aggregates token-level embeddings into fixed-size global feature vectors (**z**_*p*_ and **z**_*m*_) while excluding padded positions.

### Limitations of the study

This work focuses on DTI prediction by integrating multi-modal drug representations, sequence-level protein features, and pooled global representations from pretrained encoders. Although SF-DTI improves interaction-aware representation learning across multiple evaluation settings, it does not explicitly incorporate 3D structural information or continuous binding affinity modeling. In particular, threshold-based conversion of affinity-related datasets into binary interaction labels enables direct comparison with classification-based DTI benchmarks, but it inevitably discards part of the continuous binding-strength information and treats interactions with different affinities as equivalent within the same class. Therefore, results obtained under this protocol should be interpreted as binary interaction discrimination performance rather than quantitative binding-affinity prediction. Future work will explore structure-aware representations, affinity-aware objectives, and multi-task formulations that jointly model binary interaction labels and continuous affinity scores to further improve fine-grained interaction modeling.

## Resource availability

### Lead contact

Requests for further information and resources should be directed to and will be fulfilled by the lead contact, Xiaolan Xie (xie_xiao_lan@foxmail.com).

### Materials availability

This study did not generate new, unique reagents.

### Data and code availability

• All data supporting the findings of this study are available in the article and its supplemental information. Public benchmark datasets and external resources used in this work are listed in the key resources table. All original data reported in the manuscript will be shared by the lead contact upon request.
• All original code, including source code, data-processing scripts, split files, and analysis scripts, has been deposited on GitHub and is publicly available as of the date of publication at https://github.com/wbw176-netizen/SF-DTI.
• Any additional information required to reanalyze the data reported in this study is available from the lead contact upon request.

## Acknowledgments

This work was supported by the 10.13039/501100012166National Key R&D Program of China (2023YFC3503400 and 2022YFC3400400), the 10.13039/501100001809NSFC-10.13039/501100006469FDCT Grants (62361166662), the 10.13039/501100001809National Natural Science Foundation of China (62262011), the Innovative Research Group Project of Hunan Province (2024JJ1002), the Hunan Science and Technology Innovation Plan (2025ZYJ003), and the Key R&D Program of Hunan Province (2023GK2004, 2023SK2059, and 2023SK2060).

## Author contributions

B.W., conceptualization, methodology, software, validation, formal analysis, visualization, writing – original draft; X.X., software, validation, writing – review and editing, funding acquisition; H.Z., methodology, writing – review and editing, investigation, data curation; S.P., writing – review and editing, supervision, project administration, funding acquisition. All authors reviewed the manuscript.

## Declaration of interests

The authors declare no competing interests.

## STAR★Methods

### Key resources table

**Table:** 

REAGENT or RESOURCE	SOURCE	IDENTIFIER
**Deposited data**		

BindingDB benchmark dataset	Liu et al.^54^	https://www.bindingdb.org/
BioSNAP drug–target interaction benchmark dataset	Zitnik et al.^55^	http://snap.stanford.edu/biodata
DrugBank benchmark dataset	Wishart et al.^37^	https://go.drugbank.com/
Human drug–target interaction benchmark dataset	Liu et al.^56^	https://doi.org/10.1093/bioinformatics/btv256
C. elegans drug–target interaction benchmark dataset	Tsubaki et al.^57^	https://doi.org/10.1093/bioinformatics/bty535
KIBA benchmark dataset	Tang et al.^58^	https://doi.org/10.1021/ci400709d
SF-DTI preprocessed benchmark datasets	This paper	Google Drive
Protein Data Bank structures	Berman et al.^45^	https://www.rcsb.org/

**Software and algorithms**		

ChemBERTa-77M-MLM molecular encoder	Chithrananda et al.^59^	https://huggingface.co/DeepChem/ChemBERTa-77M-MLM
ESM-2 t33_650M_UR50D protein encoder	Lin et al.^23^	https://huggingface.co/facebook/esm2_t33_650M_UR50D
ProtT5-XL-UniRef50 protein encoder	Elnaggar et al.^24^	https://huggingface.co/Rostlab/prot_t5_xl_uniref50
PyTorch 2.2.2	PyTorch	https://pytorch.org/
Deep Graph Library (DGL) 2.0.0	DGL	https://www.dgl.ai/
DGL-LifeSci 0.3.2	DGL-LifeSci	https://github.com/awslabs/dgl-lifesci
SciPy 1.12.0	SciPy	https://docs.scipy.org/doc/scipy-1.12.0/
RDKit 2024.03.5	Landrum et al.^52^	https://doi.org/10.5281/zenodo.12782092
Uniform Manifold Approximation and Projection (UMAP)	McInnes et al.^36^	https://doi.org/10.21105/joss.00861
Molecular Operating Environment (MOE) 2024.06	Chemical Computing Group ULC^53^	https://www.chemcomp.com/release_notes/moe202406/rnotes.htm
SF-DTI source code, data-processing scripts, split files, and analysis resources	This paper	https://github.com/wbw176-netizen/SF-DTI

### Experimental model and study participant details

This study does not involve human participants, animal subjects, cell lines, or experimental model systems. All experiments are computational analyses based on publicly available drug–target interaction datasets.

### Method details

#### Datasets

We evaluated SF-DTI on six publicly available benchmark datasets, including BindingDB,^54^ BioSNAP,^55^ DrugBank,^37^ Human,^56^ KIBA,^58^ and C. *elegans*.^57^ Table 12 All datasets were formulated as drug–target interaction classification tasks. BindingDB contains experimentally measured drug–target binding records, while DrugBank is a curated DTI benchmark constructed from DrugBank annotations. BioSNAP is a balanced benchmark derived from DrugBank, in which positive interactions correspond to validated DTIs and negative interactions are sampled from previously unobserved drug–target pairs. Human and C.elegans were used with their released binary interaction labels. For all datasets, we used the benchmark-defined positive and negative labels without additional negative resampling, and the train/validation/test partitions were kept fixed across SF-DTI and all compared methods.

**Table:** Statistics of six DTI datasets

Dataset	Drugs	Proteins	Positive	Negative	Total
BindingDB	14,643	2,623	20,674	28,525	49,199
BioSNAP	4,510	2,181	13,634	13,830	27,464
DrugBank	4,254	6,645	17,511	17,511	35,022
KIBA	2,068	225	22,154	94,196	116,350
Human	2,726	2,001	3,364	3,364	6,728
*C. elegans*	1,767	1,876	3,893	3,893	7,786

KIBA integrates multiple biochemical measurements, including *K*_*i*_, *K*_*d*_, and IC_50_, into a unified kinase–inhibitor bioactivity score. To maintain a consistent classification-based evaluation protocol across all benchmarks, we converted the continuous KIBA scores into binary interaction labels using a threshold of 12.1, as adopted in previous DTI classification studies.^60^ Specifically, drug–target pairs with KIBA scores greater than 12.1 were labeled as positive interactions, whereas pairs with scores less than or equal to 12.1 were labeled as negative interactions. The resulting binary KIBA benchmark was then used for AUROC/AUPRC-based DTI classification evaluation in the same way as the other datasets. This setting supports direct comparison under a unified binary prediction framework, while the continuous affinity information in the original KIBA scores is not modeled as a regression target in this study.

#### Problem formulation

In DTI prediction, the fundamental task is to determine whether a given drug compound will interact with a specific target protein. Let $D=\left\{d_{1},\ldots ,d_{M}\right\}$ denote the set of drug compounds and $P=\left\{p_{1},\ldots ,p_{N}\right\}$ denote the set of target proteins. Historically, drugs are often represented by 1D sequences such as the Simplified Molecular Input Line Entry System (SMILES). While SMILES strings allow efficient processing via language models to capture deep chemical contexts, relying solely on 1D sequences may lead to the loss of crucial 2D topological structures. To overcome this and fully capture molecular properties, each drug $d_{i}\in D$ in our framework is characterized by a dual representation: a molecular graph $G_{i}=\left(V_{i},E_{i}\right)$ derived from its SMILES to capture spatial connectivity (where $V_{i}$ and $E_{i}$ denote the sets of atoms and chemical bonds), and a semantic embedding $x_{d,i}\in R^{d_{\mathrm{chem}}}$ obtained from a pre-trained molecular language model to capture global chemical semantics. Similarly, each protein $p_{j}\in P$ is fundamentally represented by an amino acid sequence $S_{j}$. To extract its rich biochemical semantics, $S_{j}$ is processed into a high-dimensional continuous embedding $x_{p,j}\in R^{d_{\mathrm{prot}}}$ via pre-trained protein language models (PLMs). Given these heterogeneous representations, DTI prediction aims to learn a mapping function F:D×P→[0,1], where the output ${\hat{y}}_{ij}$ represents the predicted interaction probability between *d*_*i*_ and *p*_*j*_. We formulate this as a binary classification task supervised by a ground-truth label matrix *Y* ∈ {0,1}^*M*×*N*^, where *y*_*ij*_ = 1 indicates an experimentally observed or benchmark-defined interaction, and *y*_*ij*_ = 0 otherwise.

#### SF-DTI architecture

As illustrated in Figure 4, the SF-DTI framework orchestrates drug–target interaction prediction through a progressively refined geometric alignment process spanning three principal stages. First, in the Heterogeneous Feature Encoding & Alignment stage, we process molecular graphs via a 3-layer Graph Convolutional Network (GCN) and integrate them with pre-extracted global chemical and protein language-model vectors obtained by masked mean pooling. These diverse modalities are geometrically aligned using the Subspace-Constrained Mutual Alignment Fusion (SCMAF) and Multi-Encoder Protein Mutual Interaction (MPMI) modules. Next, the aligned global features enter the Cascaded Cross-Modal Signal Propagation Fusion (CMSPF) module, which employs an iterative, energy-guided refinement mechanism to update interaction signals across modality-level representations. Finally, the Multi-Path Residual Classifier (MPRC) aggregates these refined representations through a dual-path residual decoding scheme to generate the final interaction probability.

#### Pre-extracted feature pipeline

To balance computational efficiency with representation capability, we adopt an offline feature extraction strategy, as illustrated in Figure 5. Let $X=\left\{x_{1},x_{2},\ldots ,x_{L}\right\}$ denote an input sequence, which can be either a protein sequence or a molecular SMILES string. We utilize state-of-the-art pretrained encoders, denoted as Φ(⋅), to extract context-aware embeddings. Specifically, ESM-2^23^ and ProtT5^24^ are used for proteins, while ChemBERTa^59^ is used for molecular SMILES.

Given an input X, the encoder outputs a hidden-state matrix

(Equation 1) H=Φ(X)

where $H\in R^{L^{\prime }\times d}$, *L*′ denotes the padded sequence length, and *d* is the hidden dimension.

To obtain a compact fixed-size representation, we apply masked mean pooling:

(Equation 2) $$z=\frac{1}{\sum_{\ell =1}^{L^{\prime }}m_{\ell }}\overset{L^{\prime }}{\underset{\ell =1}{\sum }}m_{\ell }h_{\ell }$$

where *m*_*ℓ*_ ∈ {0, 1} is the mask value indicating whether the *ℓ*-th position is valid, and **h**_*ℓ*_ is the *ℓ*-th row of **H**. This operation excludes padded positions and normalizes the aggregated representation by the actual sequence length.

To reduce storage costs and I/O overhead, we pre-compute and store only the pooled global feature vectors **z**, rather than the full token-level hidden states **H**. Therefore, during downstream training, the large pretrained encoders remain frozen and offline, while only lightweight projection adapters and subsequent fusion modules are optimized. Consequently, all downstream modules, including MPMI, SCMAF, and CMSPF, operate on fixed-size global feature vectors rather than full atom-, residue-, or token-level hidden-state sequences.

#### Subspace-constrained mutual alignment fusion

As shown in Figure 4E, the Subspace-Constrained Mutual Alignment Fusion (SCMAF) module is designed to integrate heterogeneous drug representations from the graph encoder and the chemical language encoder. Let **h**_*g*_ and **h**_*c*_ denote the corresponding drug features extracted from these two encoders, respectively. The SCMAF module consists of modality-specific projection, bidirectional alignment, shared subspace projection, and residual fusion.

First, the two modality-specific features are projected into a common latent space:

(Equation 3) $$f_{g}=\phi_{g}\left(h_{g}\right),\;f_{c}=\phi_{c}\left(h_{c}\right)$$

where *ϕ*_*g*_(⋅) and *ϕ*_*c*_(⋅) denote lightweight projection networks. In implementation, these two projection networks are defined as

(Equation 4) $$\phi_{g}\left(h_{g}\right)=GELU\left(LN\left(W_{g}h_{g}+b_{g}\right)\right),\;\phi_{c}\left(h_{c}\right)=GELU\left(LN\left(W_{c}h_{c}+b_{c}\right)\right),$$

where $W_{g}\in R^{d_{f}\times d_{g}}$ and $W_{c}\in R^{d_{f}\times d_{c}}$. In the default configuration, *d*_*g*_ = 256, *d*_*c*_ = 384, and *d*_*f*_ = 256.

To encourage cross-modal semantic interaction, we perform a transport-inspired bidirectional alignment transformation:

(Equation 5) $$f_{g}^{\prime }=T\left(f_{g}\|f_{c}\right),\;f_{c}^{\prime }=T\left(f_{c}\|f_{g}\right)$$

where T(⋅) denotes a learnable alignment function and ‖ denotes feature concatenation. The alignment function is implemented as

(Equation 6) $$T\left(x\right)=W_{T}^{\left(2\right)}Dropout\left(GELU\left(LN\left(W_{T}^{\left(1\right)}x+b_{T}^{\left(1\right)}\right)\right)\right)+b_{T}^{\left(2\right)},$$

where $x\in R^{2d_{f}}$, $W_{T}^{\left(1\right)}\in R^{2d_{f}\times 2d_{f}}$, and $W_{T}^{\left(2\right)}\in R^{d_{f}\times 2d_{f}}$.

Next, the aligned features are projected into a shared informative subspace:

(Equation 7) $${\hat{f}}_{g}=P\;\psi_{g}\left(f_{g}^{\prime }\right),\;{\hat{f}}_{c}=P\;\psi_{c}\left(f_{c}^{\prime }\right)$$

where *ψ*_*g*_(⋅) and *ψ*_*c*_(⋅) are modality-specific subspace projection functions, **P** is a learnable projection operator parameterized to capture the shared informative subspace. The subspace projection functions are defined as

(Equation 8) $$\psi_{m}\left(x\right)=GELU\left(LN\left(W_{m}x+b_{m}\right)\right),\;m\in \left\{g,c\right\},$$

where $W_{m}\in R^{d_{f}\times d_{f}}$. The shared projection operator **P** is parameterized by a learnable basis matrix $B\in R^{d_{f}\times d_{f}}$.

Finally, SCMAF combines subspace-averaged information, concatenation-based fusion, and residual modality-specific features to obtain the final drug representation:

(Equation 9) $$z_{d}=LN\left(\psi \left({\hat{f}}_{g}\|{\hat{f}}_{c}\right)+\frac{1}{2}\left({\hat{f}}_{g}+{\hat{f}}_{c}\right)+f_{g}+f_{c}\right)$$

where *ψ*(⋅) denotes a lightweight fusion network and LN(⋅) denotes layer normalization. The fusion network follows the same two-layer structure as T(⋅) and maps the concatenated 2*d*_*f*_-dimensional aligned representation back to *d*_*f*_ dimensions.

By jointly performing bidirectional alignment, shared subspace projection, and residual fusion, SCMAF enhances the integration of graph-derived structural patterns and pretrained chemical semantics, leading to a more robust drug representation.

#### Multi-encoder protein mutual interaction

As shown in Figure 4F, different protein language models often capture complementary biochemical semantics. To effectively integrate the pooled global protein representations extracted from ProtT5 and ESM-2, we design a Multi-Encoder Protein Mutual Interaction (MPMI) module that performs encoder-specific projection, residual-preserving alignment, and bidirectional cross-feature interaction in a shared latent space.

Let $f_{t}\in R^{d_{t}}$ and $f_{e}\in R^{d_{e}}$ denote the pooled global protein features extracted from ProtT5 and ESM-2, respectively. Since the two encoders produce heterogeneous representations with different dimensions, we first project them into a common fusion space of dimension *d*_*f*_ using encoder-specific nonlinear adapters:

(Equation 10) $$p_{t}=\phi_{t}\left(f_{t}\right),\;p_{e}=\phi_{e}\left(f_{e}\right)$$

where *ϕ*_*t*_(⋅) and *ϕ*_*e*_(⋅) denote lightweight projection networks.

To preserve useful information from the original encoder outputs, we further employ linear residual compression branches to map the raw features into the same fusion space. The transformed and residual features are then combined by learnable scalar fusion coefficients:

(Equation 11) $${\tilde{p}}_{k}=LayerNorm\left(\alpha_{k}p_{k}+\left(1-\alpha_{k}\right)r_{k}\right),\;k\in \left\{t,e\right\}$$

where **r**_*k*_ denotes the residual-compressed feature and *α*_*k*_ is a learnable scalar parameter. Learnable scaling factors are further applied to the aligned features to adjust the contribution magnitude of each encoder.

The aligned global protein features are then fused through a shared bidirectional mutual interaction operator implemented by lightweight feature projection, bidirectional exchange, residual fusion, and normalization.

(Equation 12) $$u_{t}=Int\left({\tilde{p}}_{t},{\tilde{p}}_{e}\right),\;u_{e}=Int\left({\tilde{p}}_{e},{\tilde{p}}_{t}\right)$$

where the interaction operator is implemented as a shared lightweight bidirectional fusion block for efficient cross-feature exchange.

Finally, the two interaction outputs are combined with residual information and refined by a lightweight feedforward transformation:

(Equation 13) $$z_{p}=\psi \left(LayerNorm\left(u_{t}+u_{e}+\lambda \left({\tilde{p}}_{t}+{\tilde{p}}_{e}\right)\right)\right)$$

where *λ* is a residual weighting factor and *ψ*(⋅) denotes a linear transformation followed by normalization and nonlinear activation.

By combining encoder-specific projection, residual-preserving alignment, and bidirectional interaction, MPMI enables effective fusion of complementary protein semantics from multiple pretrained encoders while maintaining a lightweight and efficient architecture.

#### Cascaded cross-modal signal propagation fusion

As shown in Figure 4B, the Cascaded Cross-Modal Signal Propagation Fusion (CMSPF) module is introduced after SCMAF and MPMI to refine the aligned drug-side and protein-side representations. At this stage, each drug–target pair is represented by compact global embeddings, and the objective is no longer to build token-level or residue-level attention maps because the pretrained encoder outputs have already been pooled into fixed-size global vectors. A conventional cross-attention or gated-fusion block therefore mainly performs a one-step reweighting of the available global features. However, DTI prediction often requires the model to gradually calibrate how much information should be taken from drug-side structural/semantic cues, protein-side context, and their emerging interaction representation.

To address this problem, CMSPF treats cross-modal fusion as an iterative stream-level refinement process. It maintains separate drug, protein, and shared interaction streams, and introduces a learnable semantic basis as a common coordinate space for comparing their current response strengths. At each cascade layer, the response magnitudes of the drug, protein, and cross-modal streams are normalized into routing coefficients, which determine how information is propagated and updated in the next refinement step. In this way, CMSPF performs pair-specific signal routing across multiple cascade layers, rather than applying a single static gate or a one-shot cross-attention operation.

1. **Shared Subspace Initialization.** Before the cascade begins, a shared communication channel is established. Let $H_{d}^{\left(0\right)}$ and $H_{p}^{\left(0\right)}$ denote the initial drug and protein features, respectively. We construct an initial shared representation $H_{s}^{\left(0\right)}$ via a gated projection mechanism. Specifically, the raw features are first concatenated and projected into a latent interaction space:

(Equation 14) $$Z_{\mathrm{raw}}=\left[H_{d}^{\left(0\right)}\|H_{p}^{\left(0\right)}\right],\;H_{s}^{\mathrm{proj}}=\phi_{\mathrm{mlp}}\left(Z_{\mathrm{raw}}\right)$$

where *ϕ*_*mlp*_(⋅) denotes a multi-layer perceptron. To suppress irrelevant noise at the initialization stage, we further introduce an adaptive gate **g** = *σ*(**W**_*g*_**Z**_*raw*_) and obtain

(Equation 15) $$H_{s}^{\left(0\right)}=g⊙H_{s}^{\mathrm{proj}}$$

The resulting $H_{s}^{\left(0\right)}$ serves as the shared anchor for subsequent cross-modal propagation.

2. **Energy-Guided Signal Routing.** At each cascade layer *l*, CMSPF estimates the relative informativeness of different streams and dynamically routes information accordingly. We define signal energy as the magnitude of feature responses projected onto a shared semantic basis **V**_*sem*_:

(Equation 16) $$E_{d}^{\left(l\right)}=\|H_{d}^{\left(l\right)}V_{\mathrm{sem}}^{⊤}{\|}_{2},\;E_{p}^{\left(l\right)}=\|H_{p}^{\left(l\right)}V_{\mathrm{sem}}^{⊤}{\|}_{2}$$

In parallel, a cross-modal interaction signal $S_{\mathrm{cross}}^{\left(l\right)}$ is computed through a joint transformation, with corresponding energy $E_{\mathrm{cross}}^{\left(l\right)}$. Intuitively, these energy terms characterize the activation intensity of each stream. Based on them, the routing coefficients are defined as

(Equation 17) $$\alpha_{k}^{\left(l\right)}=\frac{E_{k}^{\left(l\right)}}{\sum_{j\in \left\{d,p,cross\right\}}E_{j}^{\left(l\right)}+\epsilon },\;k\in \left\{d,p,cross\right\}$$

These coefficients regulate the relative contribution of each stream, enabling the model to emphasize the most informative signal source for a given drug–target pair.

The signal energy is computed as the response magnitude in this semantic basis and is used to normalize the routing coefficients across drug, protein, and cross-modal streams. In this way, each cascade layer can redistribute information according to the current pair-specific representation state, while the semantic basis and routing transformations are learned jointly with the downstream DTI prediction objective.

3. **Conditional Modulation and Cascaded Refinement.** Given the routing coefficients, CMSPF updates the modality-specific representations through conditional modulation. The shared stream injects interaction-aware information into each modality via affine transformations with scale and shift parameters. For the drug stream, the update is written as

(Equation 18) $$H_{d}^{\left(l+1\right)}=H_{d}^{\left(l\right)}+\alpha_{d}^{\left(l\right)}\cdot F_{\mathrm{refine}}\left(H_{d}^{\left(l\right)},H_{s}^{\left(l\right)}\right)$$

Symmetric update rules are applied to $H_{p}^{\left(l+1\right)}$ and $H_{s}^{\left(l+1\right)}$. Here, $F_{\mathrm{refine}}$ denotes a lightweight refinement function that captures local interaction context efficiently. After L cascaded layers (with default *L* = 4), the refined representations $H_{d}^{\left(out\right)}$, $H_{p}^{\left(out\right)}$, and $H_{s}^{\left(out\right)}$ are obtained, encoding progressively aligned and noise-suppressed interaction semantics.

#### Multi-path residual classifier

As shown in Figure 4C, we employ a Multi-Path Residual Classifier to enhance prediction robustness through parallel feature transformations and residual integration. Let **f**_*in*_ denote the input feature vector. First, the input feature is processed by two parallel transformation branches:

(Equation 19) $$h_{\mathrm{pri}}=\sigma \left(BN\left(W_{1}f_{in}\right)\right),\;h_{\sec}=\sigma \left(BN\left(W_{2}f_{in}\right)\right)$$

where **W**_1_ and **W**_2_ are learnable projection matrices, BN(⋅) denotes batch normalization, and *σ*(⋅) denotes a non-linear activation function.

The transformed features are then concatenated with the residual input and fed into a lightweight fusion classifier:

(Equation 20) $$\hat{y}=MLP\left(\left[h_{\mathrm{pri}}\|h_{\sec}\|f_{in}\right]\right)$$

where ‖ denotes concatenation, and MLP(⋅) denotes the fusion classification block that outputs the final interaction logit. A sigmoid activation is applied to $\hat{y}$ to obtain the predicted probability.

This design jointly exploits complementary transformed representations and residual information, leading to more stable and expressive classification.

#### Training and optimization

We train SF-DTI using binary cross-entropy loss:

(Equation 21) $$L=-\frac{1}{N}\overset{N}{\underset{i=1}{\sum }}\left[y_{i}\log\left({\hat{y}}_{i}\right)+\left(1-y_{i}\right)\log\left(1-{\hat{y}}_{i}\right)\right]$$

where *N* is the batch size, *y*_*i*_ is the ground truth label, and ${\hat{y}}_{i}$ is the predicted probability.

#### Evaluation scenarios

To comprehensively assess model robustness and generalization ability, we considered four evaluation scenarios.

##### Scenario I (random split)

Each dataset was randomly divided into training, validation, and test sets with a ratio of 8:1:1. Under this setting, the distributions of drug sizes (i.e., the number of atoms) and target lengths (i.e., the number of amino acids) in the training, validation, and test sets were further analyzed using categorical scatterplots. The corresponding results are available at: https://github.com/wbw176-netizen/SF-DTI/tree/main/Distributions.

##### Scenario II (5-fold cross-validation)

All drug–target pairs were randomly partitioned into five approximately equal and non-overlapping subsets. In each fold, one subset was used for testing and the remaining four subsets were used for training. A validation subset was further split from the training portion for model selection. This process was repeated five times so that each subset served as the test set once, and the final performance was reported as the average over the 5-folds.

##### Scenario III (unseen drug and target)

We used a split protocol adapted from MolTrans.^32^ In the unseen-drug setting, 20% of drugs were selected, and all DTI pairs associated with these drugs were assigned to the test set. The remaining source pairs were further divided into training and validation sets for model selection. In the unseen-target setting, the same procedure was applied to target proteins, where 20% of target proteins were selected and all associated DTI pairs were assigned to the test set. Thus, test drugs are disjoint from the training and validation sets in the unseen-drug setting, and test proteins are disjoint from the training and validation sets in the unseen-target setting. This setting evaluates the ability of the model to generalize to interactions involving previously unseen compounds or proteins.

##### Scenario IV (cluster split)

We further used the clustering-based cross-domain evaluation protocol introduced in DrugBAN.^28^ Drugs and proteins were clustered using single-linkage clustering based on ECFP4 fingerprints and pseudo amino acid composition (PSC),^61^ respectively. Specifically, 60% of the drug clusters and 60% of the protein clusters were randomly assigned to the source domain, while the remaining clusters were assigned to the target domain. All drug–target pairs between source-domain drugs and source-domain proteins were used as labeled source-domain data. Target-domain pairs were further divided into target training and target evaluation subsets, where 80% of the target-domain pairs were treated as unlabeled samples for domain adaptation and the remaining 20% formed the labeled target-domain evaluation set. Following the original DrugBAN implementation, this target-domain evaluation set was used for model selection and performance reporting for all evaluated methods. Pairs containing a source-domain drug and a target-domain protein, or a target-domain drug and a source-domain protein, were excluded. Thus, the source and target domains contain non-overlapping drug and protein clusters and exhibit distinct entity distributions. Table 13 summarizes the resulting cluster statistics for the drug and protein entities represented in the retained Scenario IV pairs.

**Table:** Clustering statistics of the retained scenario IV subsets

Dataset	Object	Count	Clusters	Max size	Min size	Mean	Median
BindingDB	drug	12,167	2,421	597	1	5.03	1
BioSNAP	drug	4,162	2,238	283	1	1.86	1
BindingDB	protein	1,926	1,295	16	1	1.49	1
BioSNAP	protein	2,150	1,953	8	1	1.10	1

On the same clustering-based data partitions, we additionally evaluated SF-DTI using the CDAN training strategy adopted in DrugBAN.^28^ CDAN was originally introduced for conditional adversarial domain adaptation.^62^ In this setting, labeled source-domain pairs are used for supervised DTI prediction, while unlabeled target-domain training pairs are used for domain-adversarial training. The domain discriminator takes the SF-DTI interaction representation and its predicted interaction probability as input and is trained to distinguish source-domain from target-domain samples. The SF-DTI network is optimized using the supervised binary cross-entropy loss together with the adversarial domain loss.

To describe the integration of CDAN, let *F*(⋅) denote the SF-DTI feature extractor up to the interaction representation, and let *G*(⋅) denote the prediction decoder. For a source-domain pair $x_{i}^{s}$ and a target-domain pair $x_{j}^{t}$, their interaction representations are

(Equation 22) $$f_{i}^{s}=F\left(x_{i}^{s}\right),\;f_{j}^{t}=F\left(x_{j}^{t}\right),$$

and the corresponding positive-class prediction probabilities are

(Equation 23) $$p_{i}^{s}=\sigma \left(G\left(f_{i}^{s}\right)\right),\;p_{j}^{t}=\sigma \left(G\left(f_{j}^{t}\right)\right),$$

where *σ*(⋅) denotes the sigmoid function. To condition domain alignment on both the interaction representation and the predicted class distribution, we used randomized multilinear conditioning. For a source- or target-domain pair with positive-class probability *p*, let **f** denote its interaction representation and **g** = [1 − *p*, *p*] denote its predicted class-probability vector. The conditional representation was computed as

(Equation 24) $$h=\frac{\left(fR_{f}\right)⊙\left(gR_{g}\right)}{\sqrt{d_{r}}},$$

where **R**_*f*_ and **R**_*g*_ are fixed random projection matrices, ⊙ denotes element-wise multiplication, and *d*_*r*_ = 256 is the randomized conditioning dimension. The resulting conditional representation was passed to the domain discriminator through a gradient-reversal layer. During adversarial training, the discriminator is optimized to predict the domain label, whereas the SF-DTI feature extractor is optimized to reduce the distribution discrepancy between source-domain and target-domain interaction representations.

The overall objective is written as

(Equation 25) $$L=L_{\mathrm{DTI}}^{\mathrm{src}}+\eta L_{DA},$$

where $L_{\mathrm{DTI}}^{\mathrm{src}}$ is the supervised binary cross-entropy loss on labeled source-domain pairs, $L_{DA}$ is the adversarial domain loss computed from source/target domain labels, and *η* controls the contribution of domain adaptation.

For Scenarios I, III, and IV, we repeated the partitioning process five times with different random seeds and reported the average results with standard deviations. For Scenario II, the final performance was reported over the 5-folds.

#### Additional experimental procedures

##### Controlled baseline construction

The controlled baselines used the same frozen, pooled ChemBERTa, ESM-2, and ProtT5 representations as SF-DTI. Concat-MLP, bilinear fusion, cross-attention, and gated-fusion models replaced the proposed fusion architecture with simpler classifiers or alternative fusion mechanisms while retaining the same pretrained input representations. These controls excluded the GCN-derived molecular graph representation, whose contribution was evaluated separately through the component-level and fine-grained SCMAF ablations.

##### Blinded case-study construction

The blinded case studies were constructed from deduplicated DrugBank pairs. Duplicate entries with the same DrugBank ID and UniProt ID were collapsed, and the positive interaction label was retained when duplicate records had conflicting labels. For drug-centric queries, all pairs involving the query drug were excluded from the training and validation sets; analogously, all pairs involving the query target were excluded for target-centric queries. The excluded query-associated pairs constituted the blinded candidate pool, which was ranked according to the predicted interaction probabilities and evaluated against the DrugBank annotations used in this study.

##### Activation-based interpretability case selection

Six representative ligand–protein complexes from the PDB were selected to cover different protein families and binding pockets. Only X-ray structures with resolution better than 2.5 Å were considered, and the corresponding drug–target pairs were not included in the training set.

##### Computational profiling

Computational cost was evaluated on the DrugBank benchmark using the default SF-DTI configuration. We recorded the number of trainable parameters, downstream training time per epoch, inference throughput, peak training and inference GPU memory usage, and storage required for the pre-extracted features. Pretrained-model representations were extracted offline before downstream training and reused during computational profiling. Downstream training was measured with a batch size of 64 and mixed precision. The corresponding computational cost analysis is reported in Table S14.

##### Failure-case selection

For the BioSNAP and BindingDB target-domain test sets under the cluster-split setting, we selected the five negative-label pairs with the highest mean predicted probabilities and the five positive-label pairs with the lowest mean predicted probabilities across repeated runs. Predicted probabilities were summarized as mean values with standard deviations, and error categories were assigned according to the benchmark test labels.

### Quantification and statistical analysis

#### Evaluation metrics

We evaluate SF-DTI using the area under the receiver operating characteristic curve (AUROC) and the area under the precision–recall curve (AUPRC). All experiments are implemented in PyTorch 2.2.2 with DGL 2.0.0 and DGL-LifeSci 0.3.2 and conducted on NVIDIA RTX 4090 GPUs. Statistical analyses were performed using SciPy 1.12.0.

For Scenarios I, III, and IV, model performance was summarized over five independent runs with different random seeds (*n* = 5, where *n* denotes independent runs). For Scenario II, performance was summarized over the 5-folds from one 5-fold cross-validation procedure (*n* = 5, where *n* denotes folds). Results were reported as mean values with standard deviations, with the mean representing the measure of center and the standard deviation representing the measure of dispersion. Statistical significance between SF-DTI and competing methods was assessed using two-sided paired t-tests on paired AUROC and AUPRC values when repeated-run or fold-level results were available. To control the family-wise error rate arising from multiple baseline comparisons, the *p*-values were adjusted using the Holm–Bonferroni procedure separately for each dataset, evaluation metric, and evaluation protocol across the five baseline comparisons. A Holm-adjusted *p*-value below 0.05 was considered statistically significant. The corresponding statistical details are reported in the Results, the relevant figure and table legends, and the Supplemental Tables.

#### Implementation details

We first determined a stable hyperparameter configuration through preliminary experiments on the Human dataset, and then fixed the same configuration for all remaining datasets unless otherwise specified. This setting avoids dataset-specific hyperparameter tuning and provides a more consistent evaluation across benchmarks. Unless otherwise specified, the main hyperparameters were set as follows: the maximum number of epochs was 100, the batch size was 64, the initial learning rate was 5 × 10^−5^, the learning-rate decay factor was 0.7, the weight decay was 1 × 10^−4^, the node input embedding dimension was 256, the embedding dimension was 256, the number of cascaded layers was 4, the number of heads was 4, and the hidden dimension was 768. For detailed hyperparameters, please refer to Table S13 in the Supplemental Materials.

All controlled experiments used the same dataset-specific training, validation, and test splits as SF-DTI, with model checkpoints selected according to validation AUROC before test-set evaluation.

## Supplemental information

Document S1. Tables S1–S16 and supplemental references
