# Supplementary material for: Multiscale cross modal fusion and cascaded signal propagation for drug target interaction prediction
Source: iScience. 2026 Sep 22;29(10):117576. doi: 10.1016/j.isci.2026.117576 (PMC13632212; doi:10.1016/j.isci.2026.117576)
Supplement: Supplementary file 1 [file mmc1.pdf]

iScience, Volume *29*

Supplemental information

Multiscale cross modal fusion and cascaded signal propagation for drug target interaction prediction

Bowen Wang, Xiaolan Xie, Haitao Zou, and Shaoliang Peng

Supplemental Information

Table S1: Paired statistical tests comparing SF-DTI with baseline methods based on AUROC. Holm–Bonferroni-adjusted p-values are reported.

Dataset	Baseline	Random split			5-fold cross-validation		
		Difference	t-value	Holm-adjusted p-value	Difference	t-value	Holm-adjusted p-value
BindingDB	BINDTI	0.0114	3.0887	0.0732	0.0071	4.6963	0.0093
	DrugBAN	0.0080	3.7991	0.0574	0.0067	6.5745	0.0083
	MADTI	0.0054	4.5661	0.0412	0.0066	12.6032	0.0011
	WDGBANDTI	0.0039	2.0258	0.1127	0.0048	6.3406	0.0083
	MMDG-DTI	0.0104	5.4324	0.0279	0.0113	7.2780	0.0076
BioSNAP	BINDTI	0.0444	12.9430	< 0.001	0.0446	30.4615	< 0.001
	DrugBAN	0.0526	11.1266	< 0.001	0.0435	19.3500	< 0.001
	MADTI	0.0473	11.8641	< 0.001	0.0469	25.1612	< 0.001
	WDGBANDTI	0.0488	12.0491	< 0.001	0.0402	26.2762	< 0.001
	MMDG-DTI	0.0333	22.3387	< 0.001	0.0312	25.2971	< 0.001
DrugBank	BINDTI	0.0748	50.5765	< 0.001	0.0627	47.7759	< 0.001
	DrugBAN	0.0692	41.0724	< 0.001	0.0636	21.6232	< 0.001
	MADTI	0.0702	25.4930	< 0.001	0.0723	25.0925	< 0.001
	WDGBANDTI	0.0688	26.0863	< 0.001	0.0644	24.9763	< 0.001
	MMDG-DTI	0.0509	38.4120	< 0.001	0.0436	50.4613	< 0.001
Human	BINDTI	0.0005	0.2788	1.0000	0.0052	3.8515	0.0548
	DrugBAN	0.0017	1.0335	1.0000	0.0064	6.4399	0.0124
	MADTI	0.0048	2.4172	0.3649	0.0091	6.7669	0.0124
	WDGBANDTI	0.0019	0.9301	1.0000	0.0063	3.7560	0.0548
	MMDG-DTI	0.0000	0.0437	1.0000	-0.0004	-0.2069	0.8462
KIBA	BINDTI	0.0149	8.8620	0.0045	0.0135	28.9680	< 0.001
	DrugBAN	0.0229	8.8198	0.0045	0.0258	9.3129	0.0030
	MADTI	0.0114	6.6933	0.0052	0.0172	8.7525	0.0030
	WDGBANDTI	0.0143	8.5886	0.0045	0.0134	7.1035	0.0041
	MMDG-DTI	0.0065	3.9686	0.0166	0.0115	4.0959	0.0149
C.elegans	BINDTI	0.0046	4.2892	0.0510	0.0041	3.5796	0.0464
	DrugBAN	0.0044	4.0977	0.0510	0.0043	8.2444	0.0047
	MADTI	0.0120	13.7139	< 0.001	0.0135	15.5511	< 0.001
	WDGBANDTI	0.0042	3.1198	0.0711	0.0066	4.9565	0.0232
	MMDG-DTI	-0.0015	-1.7616	0.1529	-0.0007	-1.3465	0.2494

P-values were adjusted using the Holm–Bonferroni procedure separately for each dataset, evaluation metric, and evaluation protocol across the five baseline comparisons. A Holm-adjusted p-value below 0.05 was considered statistically significant.

Table S2: Paired statistical tests comparing SF-DTI with baseline methods based on AUPRC. Holm–Bonferroni-adjusted p-values are reported.

**Dataset**	**Baseline**	**Random split**			**5-fold cross-validation**		
		**Difference**	**t-value**	**Holm-adjusted p-value**	**Difference**	**t-value**	**Holm-adjusted p-value**
BindingDB	BINDTI	0.0155	2.9081	0.1313	0.0077	3.3728	0.1119
	DrugBAN	0.0093	3.2676	0.1234	0.0040	4.7157	0.0460
	MADTI	0.0050	2.7531	0.1313	0.0041	2.5632	0.1249
	WDGBANDTI	0.0047	1.4921	0.2100	0.0014	1.0955	0.3348
	MMDG-DTI	0.0115	4.5361	0.0526	0.0094	3.2729	0.1119
BioSNAP	BINDTI	0.0376	12.1516	0.0013	0.0373	24.3783	< 0.001
	DrugBAN	0.0455	10.2571	0.0015	0.0362	14.4481	< 0.001
	MADTI	0.0503	7.0382	0.0030	0.0459	41.2788	< 0.001
	WDGBANDTI	0.0459	7.7568	0.0030	0.0339	14.2430	< 0.001
	MMDG-DTI	0.0279	11.8187	0.0013	0.0246	13.9288	< 0.001
DrugBank	BINDTI	0.0712	39.2652	< 0.001	0.0564	20.3311	< 0.001
	DrugBAN	0.0677	29.4277	< 0.001	0.0583	18.2679	< 0.001
	MADTI	0.0743	25.0746	< 0.001	0.0650	29.1321	< 0.001
	WDGBANDTI	0.0708	26.3182	< 0.001	0.0557	23.3797	< 0.001
	MMDG-DTI	0.0529	22.6447	< 0.001	0.0386	34.3608	< 0.001
Human	BINDTI	-0.0003	-0.1630	1.0000	0.0045	4.0345	0.0424
	DrugBAN	0.0012	0.6408	1.0000	0.0066	5.3955	0.0228
	MADTI	0.0047	1.6373	0.8845	0.0107	7.6509	0.0078
	WDGBANDTI	0.0018	0.6690	1.0000	0.0095	4.1625	0.0424
	MMDG-DTI	-0.0019	-0.9864	1.0000	-0.0016	-0.9818	0.3818
KIBA	BINDTI	0.0445	10.1861	0.0019	0.0356	25.1396	< 0.001
	DrugBAN	0.0643	10.4420	0.0019	0.0693	10.8446	0.0016
	MADTI	0.0357	7.3420	0.0037	0.0432	7.9100	0.0041
	WDGBANDTI	0.0423	12.4429	0.0012	0.0345	7.6596	0.0041
	MMDG-DTI	0.0099	2.7615	0.0508	0.0261	4.5063	0.0108
C.elegans	BINDTI	0.0041	8.8358	0.0045	0.0037	3.7307	0.0609
	DrugBAN	0.0028	3.1322	0.1053	0.0039	33.2001	< 0.001
	MADTI	0.0190	7.7644	0.0059	0.0130	11.7149	0.0012
	WDGBANDTI	0.0050	1.7437	0.1562	0.0103	3.7058	0.0609
	MMDG-DTI	-0.0018	-2.4999	0.1335	-0.0008	-1.1899	0.2999

P-values were adjusted using the Holm–Bonferroni procedure separately for each dataset, evaluation metric, and evaluation protocol across the five baseline comparisons. A Holm-adjusted p-value below 0.05 was considered statistically significant.

Table S3: Comparison of standard clustering-based SF-DTI and its auxiliary CDAN variant in Scenario IV.

**Dataset**	**Variant**	**AUROC**	**AUPRC**
BindingDB	SF-DTI	0.6765±0.0232	0.5969±0.0427
BindingDB	$\text{SF-DTI}_{\text{CDAN}}$	0.6832±0.0157	0.6127±0.0120
BioSNAP	SF-DTI	0.7917±0.0073	0.8004±0.0095
BioSNAP	$\text{SF-DTI}_{\text{CDAN}}$	0.8032±0.0060	0.8130±0.0048

Table S4: Ablation experiments on the main modules of SF-DTI.

Method variant	BindingDB		BioSNAP		DrugBank		Human		KIBA		C.elegans	
	AUROC	AUPRC	AUROC	AUPRC	AUROC	AUPRC	AUROC	AUPRC	AUROC	AUPRC	AUROC	AUPRC
SF-DTI (Full)	0.9695±0.0020	0.9601±0.0010	0.9479±0.0010	0.9466±0.0012	0.9437±0.0010	0.9429±0.0010	0.9934±0.0010	0.9937±0.0020	0.9461±0.0013	0.8458±0.0014	0.9960±0.0009	0.9963±0.0010
w/o MPMI	0.9642±0.0007	0.9465±0.0021	0.9413±0.0010	0.9318±0.0038	0.9358±0.0023	0.9329±0.0023	0.9916±0.0017	0.9931±0.0016	0.9397±0.0038	0.8323±0.0068	0.9931±0.0023	0.9939±0.0020
w/o SCMAF	0.9633±0.0007	0.9503±0.0025	0.9427±0.0015	0.9423±0.0023	0.9408±0.0013	0.9357±0.0015	0.9895±0.0006	0.9907±0.0008	0.9417±0.0038	0.8366±0.0069	0.9915±0.0019	0.9891±0.0021
w/o CMSPF	0.9652±0.0014	0.9475±0.0021	0.9429±0.0009	0.9350±0.0042	0.9373±0.0025	0.9324±0.0056	0.9930±0.0009	0.9936±0.0008	0.9409±0.0019	0.8347±0.0041	0.9947±0.0025	0.9930±0.0031

Table S5: Controlled baselines using the same pretrained representations as SF-DTI. The dual protein setting uses both ESM-2 and ProtT5 features together with ChemBERTa drug features[1–3].

Control model	BindingDB		BioSNAP		DrugBank		Human		KIBA		C.elegans	
	AUROC	AUPRC	AUROC	AUPRC	AUROC	AUPRC	AUROC	AUPRC	AUROC	AUPRC	AUROC	AUPRC
Concat-MLP	0.9560±0.0012	0.9372±0.0007	0.9294±0.0015	0.9255±0.0006	0.9240±0.0024	0.9193±0.0034	0.9759±0.0023	0.9771±0.0039	0.9190±0.0020	0.7700±0.0023	0.9833±0.0010	0.9809±0.0012
Bilinear	0.9625±0.0016	0.9463±0.0041	0.9349±0.0008	0.9297±0.0009	0.9324±0.0015	0.9246±0.0027	0.9797±0.0028	0.9797±0.0044	0.9225±0.0021	0.7836±0.0071	0.9901±0.0022	0.9900±0.0026
Cross-attention	0.9611±0.0046	0.9424±0.0039	0.9311±0.0014	0.9284±0.0037	0.9274±0.0040	0.9214±0.0014	0.9818±0.0018	0.9807±0.0023	0.9318±0.0021	0.8031±0.0034	0.9874±0.0024	0.9860±0.0042
Gated fusion	0.9609±0.0018	0.9422±0.0016	0.9360±0.0004	0.9315±0.0034	0.9306±0.0030	0.9280±0.0038	0.9832±0.0029	0.9813±0.0079	0.9300±0.0013	0.8005±0.0050	0.9867±0.0010	0.9861±0.0020

Table S6: Single-encoder and dual-encoder protein controls under the Concat-MLP architecture. This table quantifies the contribution of combining ESM-2 and ProtT5 protein representations. The ESM-2+ProtT5 row is the same dual-encoder Concat-MLP control reported in Supplementary Table S5 and is included here for direct comparison with the single-encoder settings.

Protein input	BindingDB		BioSNAP		DrugBank		Human		KIBA		C.elegans	
	AUROC	AUPRC	AUROC	AUPRC	AUROC	AUPRC	AUROC	AUPRC	AUROC	AUPRC	AUROC	AUPRC
ESM-2	0.9542±0.0008	0.9342±0.0014	0.9265±0.0016	0.9249±0.0012	0.9186±0.0009	0.9141±0.0031	0.9757±0.0015	0.9780±0.0014	0.9043±0.0083	0.7365±0.0200	0.9814±0.0007	0.9785±0.0018
ProtT5	0.9494±0.0016	0.9275±0.0036	0.9304±0.0019	0.9300±0.0010	0.9250±0.0029	0.9195±0.0023	0.9829±0.0004	0.9838±0.0004	0.9172±0.0008	0.7697±0.0008	0.9876±0.0005	0.9862±0.0010
ESM-2+ProtT5	0.9560±0.0012	0.9372±0.0007	0.9294±0.0015	0.9255±0.0006	0.9240±0.0024	0.9193±0.0034	0.9759±0.0023	0.9771±0.0039	0.9190±0.0020	0.7700±0.0023	0.9833±0.0010	0.9809±0.0012

Table S7: Fine-grained ablation of MPMI, SCMAF, and CMSPF subcomponents.

Ablation variant	BindingDB		BioSNAP		DrugBank		Human		KIBA		C.elegans	
	AUROC	AUPRC	AUROC	AUPRC	AUROC	AUPRC	AUROC	AUPRC	AUROC	AUPRC	AUROC	AUPRC
MPMI w/o bidirectional interaction	0.9658±0.0009	0.9505±0.0022	0.9459±0.0006	0.9413±0.0042	0.9448±0.0017	0.9446±0.0013	0.9938±0.0008	0.9941±0.0007	0.9401±0.0032	0.8334±0.0061	0.9928±0.0032	0.9931±0.0027
MPMI projection only	0.9659±0.0004	0.9508±0.0020	0.9437±0.0013	0.9423±0.0034	0.9425±0.0029	0.9391±0.0032	0.9922±0.0026	0.9919±0.0025	0.9368±0.0043	0.8265±0.0074	0.9944±0.0014	0.9950±0.0013
SCMAF w/o shared subspace	0.9659±0.0003	0.9489±0.0034	0.9469±0.0007	0.9447±0.0027	0.9420±0.0013	0.9388±0.0020	0.9933±0.0006	0.9935±0.0006	0.9413±0.0035	0.8352±0.0066	0.9931±0.0018	0.9918±0.0010
SCMAF average fusion	0.9622±0.0020	0.9491±0.0016	0.9452±0.0014	0.9454±0.0042	0.9432±0.0016	0.9408±0.0023	0.9931±0.0013	0.9934±0.0012	0.9395±0.0038	0.8320±0.0065	0.9957±0.0002	0.9959±0.0002
SCMAF projection only	0.9663±0.0010	0.9503±0.0015	0.9458±0.0022	0.9427±0.0018	0.9450±0.0013	0.9420±0.0009	0.9924±0.0006	0.9925±0.0009	0.9382±0.0041	0.8291±0.0072	0.9925±0.0020	0.9924±0.0031
CMSPF fixed gate	0.9666±0.0007	0.9517±0.0010	0.9435±0.0009	0.9385±0.0015	0.9426±0.0012	0.9368±0.0027	0.9933±0.0012	0.9936±0.0010	0.9403±0.0016	0.8332±0.0059	0.9944±0.0028	0.9943±0.0038
CMSPF learned scalar gate	0.9662±0.0020	0.9509±0.0031	0.9429±0.0009	0.9374±0.0068	0.9409±0.0037	0.9337±0.0084	0.9952±0.0005	0.9953±0.0005	0.9402±0.0021	0.8309±0.0023	0.9942±0.0022	0.9941±0.0023

Table S8: Impact of Cascade Depth in CMSPF Module. Increasing depth generally improves performance, with 4 cascades offering the best trade-off.

Cascade depth	BindingDB		BioSNAP		DrugBank		Human		KIBA		C.elegans	
	AUROC	AUPRC	AUROC	AUPRC	AUROC	AUPRC	AUROC	AUPRC	AUROC	AUPRC	AUROC	AUPRC
1 Cascade	0.9668±0.0003	0.9522±0.0029	0.9421±0.0030	0.9368±0.0058	0.9402±0.0022	0.9343±0.0028	0.9931±0.0006	0.9936±0.0005	0.9392±0.0013	0.8251±0.0029	0.9954±0.0004	0.9945±0.0024
2 Cascades	0.9664±0.0012	0.9517±0.0019	0.9443±0.0016	0.9404±0.0019	0.9424±0.0010	0.9374±0.0008	0.9919±0.0002	0.9927±0.0009	0.9408±0.0009	0.8269±0.0014	0.9949±0.0008	0.9950±0.0016
3 Cascades	0.9647±0.0010	0.9496±0.0031	0.9436±0.0024	0.9389±0.0069	0.9411±0.0013	0.9336±0.0047	0.9927±0.0021	0.9936±0.0020	0.9382±0.0014	0.8232±0.0032	0.9943±0.0009	0.9943±0.0024
4 Cascades (Default)	0.9695±0.0020	0.9601±0.0010	0.9479±0.0010	0.9466±0.0012	0.9437±0.0010	0.9429±0.0010	0.9934±0.0010	0.9937±0.0020	0.9461±0.0013	0.8458±0.0014	0.9960±0.0009	0.9963±0.0010

Table S9: Hyperparameter sensitivity analysis on DrugBank.

**Hyperparameter**	**Setting**	**AUROC**	**AUPRC**
Default	$L = 4$, heads=4, dim=256, $\lambda = 0.5$, lr=5×10^{-5}	0.9438±0.0021	0.9390±0.0051
Cascade depth	$L = 5$	0.9446±0.0006	0.9414±0.0003
Cascade depth	$L = 6$	0.9439±0.0015	0.9395±0.0035
Attention heads	2	0.9415±0.0002	0.9347±0.0021
Attention heads	8	0.9474±0.0010	0.9474±0.0010
Fusion dimension	128	0.9393±0.0016	0.9371±0.0006
Fusion dimension	384	0.9421±0.0013	0.9378±0.0013
Residual weight λ	0.3	0.9432±0.0003	0.9414±0.0009
Residual weight λ	0.7	0.9428±0.0021	0.9402±0.0004
Learning rate	1×10^{-5}	0.9393±0.0010	0.9361±0.0059
Learning rate	1×10^{-4}	0.9420±0.0021	0.9384±0.0026

Table S10: Blinded case-study split statistics.

**Query**	**Setting**	**Candidate pairs**	**Removed confirmed pairs**	**Non-confirmed candidates**
NADH (DB00157)	Drug-centric	145	144	1
Beta-D-Glucose (DB02379)	Drug-centric	92	90	2
FAD (DB03147)	Drug-centric	88	86	2
H1R (P35367)	Target-centric	101	101	0
ADRA1C (P35348)	Target-centric	118	115	3
ADRA2A (P08913)	Target-centric	95	95	0
BACE1 (P56817)	Target-centric	38	27	11
HTR7 (P34969)	Target-centric	48	37	11

Table S11: Top-ranked drug candidates predicted for the additional BACE1 and HTR7 target-centric case studies.

**Rank**	**BACE1 (P56817)**			**HTR7 (P34969)**		
	**DrugBank ID**	**Predicted score**	**Result**	**DrugBank ID**	**Predicted score**	**Result**
1	DB07346	0.994087	Confirmed	DB01238	0.999441	Confirmed
2	DB07736	0.980800	Confirmed	DB09304	0.999159	Confirmed
3	DB00892	0.978815	Unconfirmed	DB13345	0.999146	Confirmed
4	DB07735	0.968450	Confirmed	DB12141	0.998933	Confirmed
5	DB07573	0.963182	Confirmed	DB00458	0.998866	Confirmed
6	DB07737	0.958755	Confirmed	DB00321	0.998584	Confirmed
7	DB07110	0.892179	Confirmed	DB00502	0.998529	Confirmed
8	DB00408	0.886038	Unconfirmed	DB00988	0.998415	Confirmed
9	DB07734	0.875959	Confirmed	DB00715	0.998329	Confirmed
10	DB07738	0.854135	Confirmed	DB06148	0.998262	Confirmed

Table S12: PDB complexes used for the activation-based interpretability analysis[4].

**PDB ID**	**Protein target**	**Resolution**
1B6K	HIV-1 protease	1.85 Å
1IEP	ABL kinase domain	2.10 Å
1X70	Dipeptidyl peptidase IV	2.10 Å
3EIG	Dihydrofolate reductase	1.70 Å
5W8L	Lactate dehydrogenase A	1.95 Å
6QL2	Carbonic anhydrase II	1.30 Å

Table S13: Hyperparameters and Model Configuration Details of SF-DTI.

Category	Item	Value
**Training Hyperparameters**		
	SOLVER.MAX_EPOCH	100
	SOLVER.BATCH_SIZE	64
	SOLVER.LR	5×10^{-5}
	SOLVER.WEIGHT_DECAY	1×10^{-4}
	SOLVER.LR_DECAY	0.7
	SOLVER.SCHEDULER	cosine
	SOLVER.FUSION_DROPOUT_RATE	0.1
	SOLVER.USE_LD	True
**Dimension Settings**		
	DRUG.NODE_IN_FEATS	75
	DRUG.HIDDEN_LAYERS	$[256, 512, 512, 256]$
	DRUG.NODE_IN_EMBEDDING	256
	PROTEIN.NUM_FILTERS	$[256, 512, 512, 256]$
	PROTEIN.EMBEDDING_DIM	256
	PROTEIN.NUM_HEAD	4
	SIGNAL.NUM_HEAD	4
	SIGNAL.EMBEDDING_DIM	256
	SIGNAL.NUM_CASCADES	4
	SIGNAL.SIGNAL_DROPOUT_RATE	0.1
	PRETRAINED.CHEMBERTA_DIM	384
	PRETRAINED.PROTT5_DIM	1024
	PRETRAINED.ESM2_DIM	1280
	DRUG_FUSION.NUM_HEAD	4
	DRUG_FUSION.DROPOUT_RATE	0.1
	PROTEIN_FUSION.NUM_HEAD	4
	PROTEIN_FUSION.DROPOUT_RATE	0.1
	DECODER.NAME	MPRC
	DECODER.IN_DIM	768
	DECODER.HIDDEN_DIM	1536
	DECODER.OUT_DIM	384
**Frozen Pretrained Encoders**		
	ChemBERTa-77M-MLM (output 384-dim)	3.4 M
	ProtT5-XL-UR50D (output 1024-dim)	2,818.8 M
	ESM-2 t33_650M_UR50D (output 1280-dim)	652.4 M
**Trainable Model Parameters**		
	SF_DTI class (models.py, 4 cascades)	38.08 M
**Model Checkpoint**		
	model.pth	$\approx$ 149 MB

Table S14: Computational cost analysis of SF-DTI on the DrugBank benchmark.

**Metric**	**Value**
Trainable parameters	38.08M
Training batch size	64
Mixed-precision training	Enabled
Estimated downstream training time per epoch	3.15 min
Offline pretrained-feature extraction	Excluded from training-time measurement
Peak GPU memory during training	8.10 GB
Inference throughput	264.69 samples/s
Peak GPU memory during inference	2.31 GB
Storage of precomputed split input files	359.38 MB

Table S15: Representative false-positive and false-negative predictions of the standard SF-DTI model on the BioSNAP target-domain test set under the cluster-split setting (Scenario IV). Sample indices are zero-based row indices in the released target-domain test file. False-positive and false-negative designations are defined relative to the benchmark test labels.

**Error type**	**Sample index**	**Drug cluster**	**Target cluster**	**Benchmark label**	**Predicted probability**
False positive	616	2082	359	0	0.9990±0.0008
False positive	76	1212	485	0	0.9941±0.0073
False positive	270	725	541	0	0.9901±0.0155
False positive	162	1082	674	0	0.9875±0.0121
False positive	466	1294	148	0	0.9839±0.0194
False negative	664	2152	695	1	0.0004±0.0002
False negative	203	173	519	1	0.0006±0.0006
False negative	80	8	1641	1	0.0026±0.0035
False negative	817	701	1789	1	0.0040±0.0023
False negative	178	790	242	1	0.0043±0.0026

Table S16: Representative false-positive and false-negative predictions of the standard SF-DTI model on the BindingDB target-domain test set under the cluster-split setting (Scenario IV). Sample indices are zero-based row indices in the released target-domain test file. False-positive and false-negative designations are defined relative to the benchmark test labels.

**Error type**	**Sample index**	**Drug cluster**	**Target cluster**	**Benchmark label**	**Predicted probability**
False positive	65	1417	598	0	0.9471±0.0291
False positive	188	731	393	0	0.9346±0.0291
False positive	322	194	955	0	0.9325±0.0441
False positive	994	1132	729	0	0.9313±0.0768
False positive	1614	194	955	0	0.9186±0.0473
False negative	1625	334	556	1	0.0212±0.0132
False negative	1686	302	1357	1	0.0234±0.0140
False negative	1304	1823	464	1	0.0243±0.0116
False negative	1495	1036	509	1	0.0368±0.0094
False negative	1204	416	509	1	0.0387±0.0286

Supplemental References

1. Chithrananda, S., G. Grand, and B. Ramsundar (2020). "ChemBERTa: Large-Scale Self-Supervised Pretraining for Molecular Property Prediction". In: *Machine Learning for Molecules Workshop at NeurIPS*.
2. Lin, Z. et al. (2023). "Evolutionary-scale prediction of atomic-level protein structure with a language model". In: *Science* 379, pp. 1123–1130. DOI: 10.1126/science.ade2574.
3. Elnaggar, A. et al. (2022). "ProtTrans: Toward Understanding the Language of Life Through Self-Supervised Learning". In: *IEEE Trans. Pattern Anal. Mach. Intell.* 44, pp. 7112–7127. DOI: 10.1109/TPAMI.2021.3095381.
4. Berman, H. et al. (2000). "The Protein Data Bank". In: *Nucleic Acids Res.* 28, pp. 235–242. DOI: 10.1093/nar/28.1.235.
